# The Performance-Result Gap in Mixed-Reality Cycling – Evidence From the Virtual Tour de France 2020 on Zwift

**DOI:** 10.3389/fphys.2022.868902

**Published:** 2022-05-13

**Authors:** Daniel Westmattelmann, Benedikt Stoffers, Marius Sprenger, Jan-Gerrit Grotenhermen, Gerhard Schewe

**Affiliations:** Center for Management, Chair of Business Administration - Organization, Personnel and Innovation, University of Münster, Münster, Germany

**Keywords:** mixed-reality, cycling, result prediction, power output, elite athletes, competition

## Abstract

**Background:** Mixed-reality sports are increasingly reaching the highest level of sport, exemplified by the first Virtual Tour de France, held in 2020. In road races, power output data are only sporadically available, which is why the effect of power output on race results is largely unknown. However, in mixed-reality competitions, measuring and comparing the power output data of all participants is a fundamental prerequisite for evaluating the athlete’s performance.

**Objective:** This study investigates the influence of different power output parameters (absolute and relative peak power output) as well as body mass and height on the results in mixed-reality competitions.

**Methods:** We scrape data from all six stages of the 2020 Virtual Tour de France of women and men and analyze it using regression analysis. Third-order polynomial regressions are performed as a cubic relationship between power output and competition result can be assumed.

**Results:** Across all stages, relative power output over the entire distance explains most of the variance in the results, with maximum explanatory power between 77% and 98% for women and between 84% and 99% for men. Thus, power output is the most powerful predictor of success in mixed-reality sports. However, the identified performance-result gap reveals that other determinants have a subordinate role in success. Body mass and height can explain the results only in a few stages. The explanatory power of the determinants considered depends in particular on the stage profile and the progression of the race.

**Conclusion:** By identifying this performance-result gap that needs to be addressed by considering additional factors like competition strategy or the specific use of equipment, important implications for the future of sports science and mixed-reality sports emerge.

## Introduction

Mixed-reality sports platforms such as Zwift allow the athletic performance provided in the real world to be transferred into a virtual space so that the participating athletes can also physically interact and even compete with each other (Speicher et al., 2019; Westmattelmann et al., 2021a). Driven by the Covid-19 pandemic, mixed-reality sports have reached the highest level of sports in a very short time, with the first-ever Virtual Tour de France held in July 2020 (Westmattelmann et al., 2021a). Both the women’s and men’s races, which were hosted on the indoor cycling platform Zwift, featured professional road cyclists (Westmattelmann et al., 2021b). In the men’s race, several “real-world” Tour de France overall winners and stage winners participated (Cyclingnews, 2020). The races gained a lot of public attention and were broadcast in over 130 countries (Cyclingnews, 2020). The fact that in December 2020, the world cycling federation (UCI) for the first time hosted the “UCI Cycling Esports World Championships” reflects the rising interest from sports organizations (UCI, 2020). In addition to participating in competitions, professional cyclists use mixed-reality sports platforms for training or social interaction (Westmattelmann et al., 2021c).

In mixed-reality cycling races, the speed of the avatar within the virtual world depends on the physical power generated (in watts), in addition to the athlete’s body mass and height (Delaney and Bromley, 2020). Cycling power is measured through a so-called smart trainer on which the athlete’s road bike is mounted. Besides physical performance, the avatar’s speed may also depend on additional factors like slipstream effects, simulated course profiles or virtual equipment. Additionally, so-called power-ups are available, whose strategic use may also affect the race results. Mixed-reality sports platforms use a number of implemented algorithms to define how athletic performance on the smart trainer is transferred to the virtual world and can be analogously compared to the laws of nature in the real world. Similarly, rolling resistance, aerodynamic drag, and the movement on mixed-reality sports platforms are also simulated according to defined rules (Kyle, 2003). In both the real world and the virtual world, knowledge of all relevant parameters, such as power output or body mass and height of the rider and course characteristics, could be used to accurately determine the speed or riding time over a defined course in individual time trials. In mass-start races, such as the (Virtual) Tour de France, the behavior of the riders and the associated interaction (e.g., slipstream) lead to an emergent system behavior that is not represented by the laws of nature (real world) or is not included in the algorithm on mixed-reality sports platforms (virtual world).

Given the complex interplay of various factors that may be critical to success in cycling races, exercise physiology research can be divided into two domains. The first domain focuses on factors that influence the physical performance of athletes in endurance sports, which in cycling is measured as absolute power in watts or relative power in watts per kilogram body weight. In their review, Jeukendrup and Martin, (2001) summarized factors that determine performance in cycling and ranked them in order of importance. According to their study, the greatest impact on cycling performance originates from the internal factor training, particularly a training program. They also valued altitude training as having a positive impact on performance. Particularly in the context of indoor cycling, the starting strategy (Mattern et al., 2001), the time of day (Atkinson et al., 2005), warm-up (Munro et al., 2017), recovery duration (Glaister et al., 2005), and precooling strategy (Quod et al., 2008) could also be considered as factors influencing performance. In cycling, the seating position (standing or seated) also influences performance (Hansen and Waldeland, 2008). Furthermore, gear ratio and pedaling cadence have a direct impact on cycling economy/efficiency (Faria et al., 2005). Finally, Atkinson et al. (2003) identified the athletes’ nutritional strategy before, during, and after a race as a determinant of performance in addition to the factors mentioned so far.

In the second domain, sports physiology literature discusses numerous factors - like physical performance delivered by the athlete - that can predict the outcome of competitions or the sporting success of an athlete (Alvero-Cruz et al., 2019; Sousa et al., 2021). Race success correlates with the peak power an athlete is able to perform in a laboratory test (Faria et al., 2005). Thus, it can be noted that the outcome of a cycling race depends on power-to-weight characteristics (Gallo et al., 2021; Lee et al., 2002). Van Erp and Sanders, (2020) show that in professional cycling races maximum mean power over shorter durations (<5 min) are higher for riders who are placed in the top-10 of a race than for riders who are not in the top-10. In addition to physiological parameters, factors such as the bike mass and body mass (both are particularly important in the mountains; Jeukendrup and Martin, 2001) as well as aerodynamic components such as body position, bike frame, and wheels can affect the competition result (Faria et al., 2005; Malizia et al., 2021). Based on their machine learning approach, Kholkine et al. (2020) assume that factors such as weather, team strategy, road conditions, or mechanical failure must be included when predicting a competition result. Another study by Van Erp et al. (2021b) showed that a cyclists’ position in the peloton could be an indicator for the result of a stage at the Tour de France. In terms of para-cyclists’ performance, Wright, (2016) also found that pacing strategy improves athletes’ performance in short time trials on cycling track.

To summarize the current state of research in the two domains, we can state that the factors determining an athlete’s physical performance are widely known, particularly in cycling. In contrast, the effect of the physical performance delivered in competition on the competition result requires further research. Studies show that several factors, such as high peak power or high power-to-weight ratio, can influence the result in cycling races. However, the physical performance was mostly determined in laboratory tests with a time lag before or after the competition (e.g., Lee et al., 2002; Babault et al., 2018; Gallo et al., 2021). Thus, it is still unclear what influence the actual power output in the competition has on the competition result. Although performance in-competition data from professional athletes are increasingly available via platforms such as Strava, the quantity of available data is still insufficient to calculate the effect on the competition result (Sanders and Heijboer, 2019; Van Erp and Sanders, 2020; Westlake, 2020; Van Erp et al., 2021a).

In mixed-reality competitions the performance data, body mass and body height of all participants are recorded and published. This happens for two reasons. First, without the disclosure of the recorded data, the athlete’s avatar would not move in the virtual world, and thus the competition would not be possible. Second, the performance data of all participants are publicly available during and after the race for all participants and spectators and are even integrated into the broadcasts to increase transparency about the race result. It was shown that the relative power output in mixed-reality races in which professional athletes participated is comparable to the relative performances in professional road races (Westmattelmann et al., 2021b). Since mixed-reality races are generally shorter than road races, the performance delivered is more similar to real-world time trials or mountain top finishes (Westmattelmann et al., 2021b). Although mixed-reality competitions publish the in-competition performance data from all professional road cyclists participating in the same competition publicly, it is unclear whether critical performances over specific time periods are particularly more relevant to race results than other performance parameters.

Regarding this research gap, the aim of this study is to analyze the effect of different performance parameters and the rider’s body mass and height on the results of mixed-reality competitions. In doing so, the following research question is addressed by analyzing in-competition data and results from the six stages of the women’s and men’s Virtual Tour de France: What influence do 1) absolute power over the entire distance, 2) relative power over the entire distance, 3) maximum relative power over 20 min, 4) maximum relative power over 5 min, 5) maximum relative power over 1 min, 6) maximum relative power over 30 s, 7) maximum relative power over 15 s, 8) body mass, and 9) body height of a rider have on the competition result (measured in riding time)?

The following section describes how the performance data for the different Virtual Tour de France stages 2020 are collected and analyzed. Subsequently, the influence of the in-competition performance parameters and the rider’s body mass and height on the result is determined via polynomial regression analyses. The findings are discussed against the background of insights from sports physiology, and practical implications for the future of (mixed-reality) sports are presented. Finally, the main insights are summarized in the conclusion.

## Materials and Methods

### Ethical Approval

This study was approved by the Ethics Committee of the School of Business & Economics of the University of Münster, Germany. Since only publicly available data from the Zwift Power platform was used, no informed consent was required from the athletes.

### Experimental Design

**FIGURE 1:**
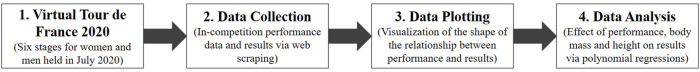
Experimental design.

Given the novelty of mixed-reality sport for exercise physiology science, we first provide an overview of the four phases of the experimental design (Figure 1).

Phase 1 marks the three weekends in July 2020 where the Virtual Tour de France took place. The performance data (absolute and relative power output) as well as body mass and height were integrated into the extensive broadcasting of the competitions and uploaded to the Zwift Power platform and are therefore publicly available, even after the end of the competitions. In phase 2, the athletes’ performance data, body mass and height, and the corresponding results of the six stages of the Virtual Tour de France for women and men were obtained using the web scraping technique from Zwift Power. In phase 3, the different performance data, body mass and height were plotted with the competition result (measured in riding time) to visualize the relationship between the determinants (power output, body mass and height) and the competition result. In Phase 4, a cubic relationship was assumed based on the visualizations in phase 3. The influence of the determinants on the competition outcome was calculated using third order polynomial regressions to account for the cubic relationship. The following subsections are organized according to each of the four phases of the experimental design.

### Virtual Tour de France

**FIGURE 2:**
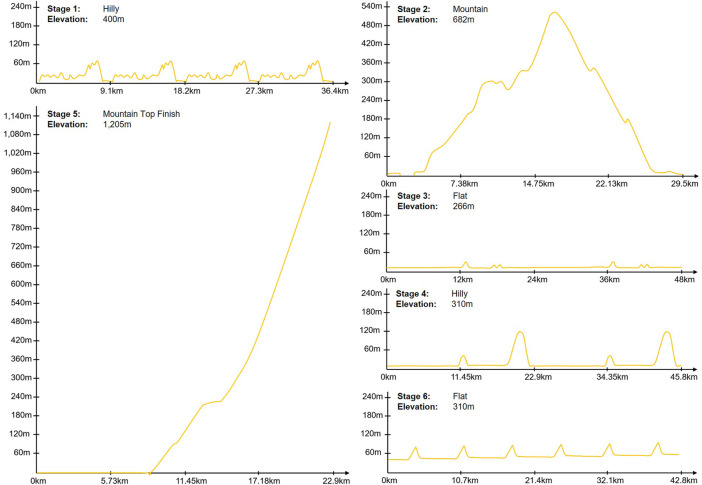
Stage profiles Virtual Tour de France. Source: www.letour.fr.

In July 2020, the cycling platform Zwift and the Amaury Sport Organisation (ASO) cooperatively hosted the first “Virtual Tour de France” (Tour de France, 2020b). In this event female and male professional cyclists competed for individual stage victories and overall team classifications in a total of six stages (Tour de France, 2020a). Unlike the real Tour de France, an annual event which is also organized by the ASO, the classifications (overall, youth, mountain, and sprint) were not awarded to individual riders but to the best teams. To have the highest chance of winning, the professional road cycling teams nominated four riders for each stage who were best suited to the different stage profiles (Schlange, 2020). Thereby, no male rider was allowed to race more than three and no female rider more than four stages in total. The different stages were won by the riders who completed the route in the shortest riding time. However, the avatar’s speed and thus the competition result also depended on the use of so-called power-ups (e.g., the aero power-up makes a rider more aerodynamic for 15 s) or riding in the slipstream. The six stages of the Virtual Tour de France are partly modeled after the real Tour de France (for example, by recreating the Mont Ventoux climb or the circuit on the Champs-Élysées in Paris, Tour de France, 2020a). The route profiles of the stages of the Virtual Tour de France are illustrated in Figure 2.

### Data Collection and Sample

To be able to analyze the influence of different performance indicators as well as body mass and height on the race results of the stages of the Virtual Tour de France, we scraped the performance data, body mass, body height, and the corresponding riding times from the platform Zwift Power (Ostanek, 2020). To do so, we used the web scraping tool Parsehub and scraped all six stages individually. Zwift Power collects different racing data for public races scheduled and performed on Zwift. After identifying the type of data relevant for our study, we used the .csv output file generated by Parsehub that contains the data to perform our analysis. For this study, the following data for all stages of the women’s and men’s races were retrieved: absolute power (watts) and relative power (w/kg) over the entire distance, as well as relative peak power over 20 min (w/kg_20m), 5 min (w/kg_5m), 1 min (w/kg_1m), 30 s (w/kg_30s), 15 s (w/kg_15s), body mass and height, average heart rate (HR_avg), and maximum heart rate (HR_max) over the entire race duration. Finally, riding times were retrieved, which are used as the dependent variable (competition success). Compared to the pure competition rankings, the metric scaling of the riding times accounts for differences between the ranks. The descriptive statistics of all athletes who finished the respective stage appear in the competition results and are shown in Tables 1 and 2. For each stage, the length, the elevation meters, and the winner’s time are also presented.

**TABLE 1:** Descriptive statistics for participants of women’s stages.

		Watts	W/kg	W/kg 20m	W/kg 5m	W/kg 1m	W/kg 30s	W/kg 15s	HR avg	HR Max	Body mass	Body height
**Stage 1** Length: 36.4 km Elevation: 400 m Time: 0:45:17	N	33	33	33	33	33	33	33	22	22	29	28
	Mean	235	4.08	4.30	4.88	5.72	6.74	8.08	172	188	58.1	167.7
	SD	30	0.47	0.43	0.35	0.61	1.09	1.94	10	9	5.3	5.6
**Stage 2** Length: 29.5 km Elevation: 682 m Time: 0:41:12	N	42	42	42	42	42	42	42	26	26	38	33
	Mean	224	4.13	4.56	5.05	5.99	6.86	8.04	172	187	54.3	166.3
	SD	26	0.52	0.47	0.62	0.85	1.35	1.87	10	10	5.4	6.2
**Stage 3** Length: 48.0 km Elevation: 266 m Time: 0:59:24	N	33	33	29	29	29	29	29	19	17	26	24
	Mean	231	3.82	4.19	4.63	5.81	6.98	8.22	171	192	61.6	170.5
	SD	32	0.47	0.36	0.35	0.76	1.30	1.95	13	11	3.9	5.4
**Stage 4** Length: 45.8 km Elevation: 310 m Time: 0:58:06	N	39	39	33	33	33	33	33	27	24	36	32
	Mean	226	3.97	4.55	5.07	6.10	7.35	8.80	170	192	57.5	168.3
	SD	32	0.53	0.38	0.43	0.57	1.24	2.05	12	10	5.5	6.3
**Stage 5** Length: 22.9 km Elevation: 1205 m Time: 0:46:21	N	33	33	33	33	33	33	33	21	21	30	28
	Mean	238	4.43	4.72	5.11	5.88	6.37	6.82	170	188	53.8	166.4
	SD	31	0.59	0.49	0.52	0.80	1.05	1.35	24	11	5.1	5.5
**Stage 6** Length: 42.8 km Elevation: 310 m Time: 0:51:44	N	29	29	29	29	29	29	29	23	23	28	26
	Mean	242	4.01	4.31	4.85	6.08	7.17	8.29	169	192	60.6	170.3
	SD	34	0.56	0.44	0.33	0.71	1.32	1.84	16	15	5.3	5.9

Note. Watts, absolute power in watts; W/kg, relative power in watt per kg bodyweight; W/kg 20m, relative peak power over 20 min; W/kg 5m, relative peak power over 5 min; W/kg 1m, relative peak power over 1 min; W/kg 30s, relative peak power over 30 s; W/kg 15s, relative peak power over 15 s; HR avg, average heartrate in beats per minute; HR max, maximum heartrate in beats per minute; body mass, rider’s body mass in kilogram; height, rider’s body height in centimeter; N, sample size; SD, standard deviation.

**TABLE 2:** Descriptive statistics for participants of men’s stages.

		Watts	W/kg	W/kg 20m	W/kg 5m	W/kg 1m	W/kg 30s	W/kg 15s	HR avg	HR max	Body mass	Body height
**Stage 1** Length: 36.4 km Elevation: 400 m Time: 0:45:17	N	24	24	24	24	24	24	24	19	19	24	20
	Mean	338	4.88	5.29	5.89	7.17	8.87	10.05	171	188	69.3	181.2
	SD	46	0.57	0.39	0.43	0.86	1.74	2.32	10	9	5.1	5.2
**Stage 2** Length: 29.5 km Elevation: 682 m Time: 0:41:12	N	25	25	25	25	25	25	25	16	16	24	18
	Mean	332	5.04	5.76	6.24	7.06	9.14	10.97	166	185	66.5	179.8
	SD	36	0.59	0.43	0.48	0.93	2.26	2.81	8	7	6.0	5.6
**Stage 3** Length: 48.0 km Elevation: 266 m Time: 0:59:24	N	20	20	18	18	18	18	18	9	8	19	13
	Mean	314	4.41	4.87	5.51	6.75	7.54	8.49	155	181	70.8	184.6
	SD	54	0.69	0.55	0.46	0.86	1.43	1.92	19	10	7.9	5.3
**Stage 4** Length: 45.8 km Elevation: 310 m Time: 0:58:06	N	27	27	23	23	23	23	23	14	12	25	21
	Mean	325	4.73	5.30	6.01	7.05	8.20	9.47	165	185	68.4	182.1
	SD	42	0.52	0.45	0.67	1.03	1.61	2.10	11	10	6.9	6.0
**Stage 5** Length: 22.9 km Elevation: 1205 m Time: 0:46:21	N	15	15	15	15	15	15	15	8	8	14	12
	Mean	334	5.18	5.72	6.09	6.65	7.03	7.53	166	186	65.5	178.0
	SD	35	0.78	0.59	0.56	0.64	0.72	0.84	16	9	7.3	6.8
**Stage 6** Length: 42.8 km Elevation: 310 m Time: 0:51:44	N	19	19	19	19	19	19	19	10	10	18	16
	Mean	330	4.67	4.98	5.53	7.14	8.11	9.11	170	189	71.4	184.1
	SD	54	0.66	0.50	0.32	1.05	1.74	2.27	11	8	4.9	5.5

Note. Watts, absolute power in watts; W/kg, relative power in watt per kg bodyweight; W/kg 20m, relative peak power over 20 min; W/kg 5m, relative peak power over 5 min; W/kg 1m, relative peak power over 1 min; W/kg 30s, relative peak power over 30 s; W/kg 15s, relative peak power over 15 s; HR avg, average heartrate in beats per minute; HR max, maximum heartrate in beats per minute; body mass, rider’s body mass in kilogram; height, rider’s body height in centimeter; N, sample size; SD, standard deviation.

The winning time in the different stages of the men’s race ranged between 41 and 59 min. Accordingly, the length of the virtual stages corresponds more to that of a time trial, which in the Virtual Tour de France was 36.8 km long, and the winning time was 55 min (Sanders and Heijboer, 2019; Procyclingstats, 2020). Women raced the same distances as men in the Virtual Tour de France, with winning times ranging from 47 to 66 min.

### Data Plotting

**FIGURE 3:**
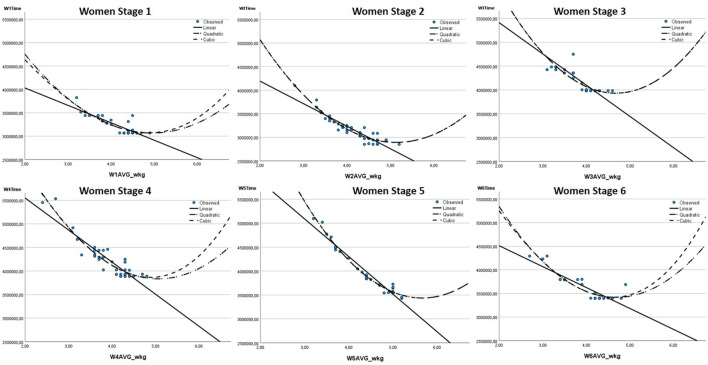
Scatterplot and fitted linear, quadratic and cubic relationship of relative power output (W/kg) and result (riding time in milliseconds) for women’s stages.

**FIGURE 4:**
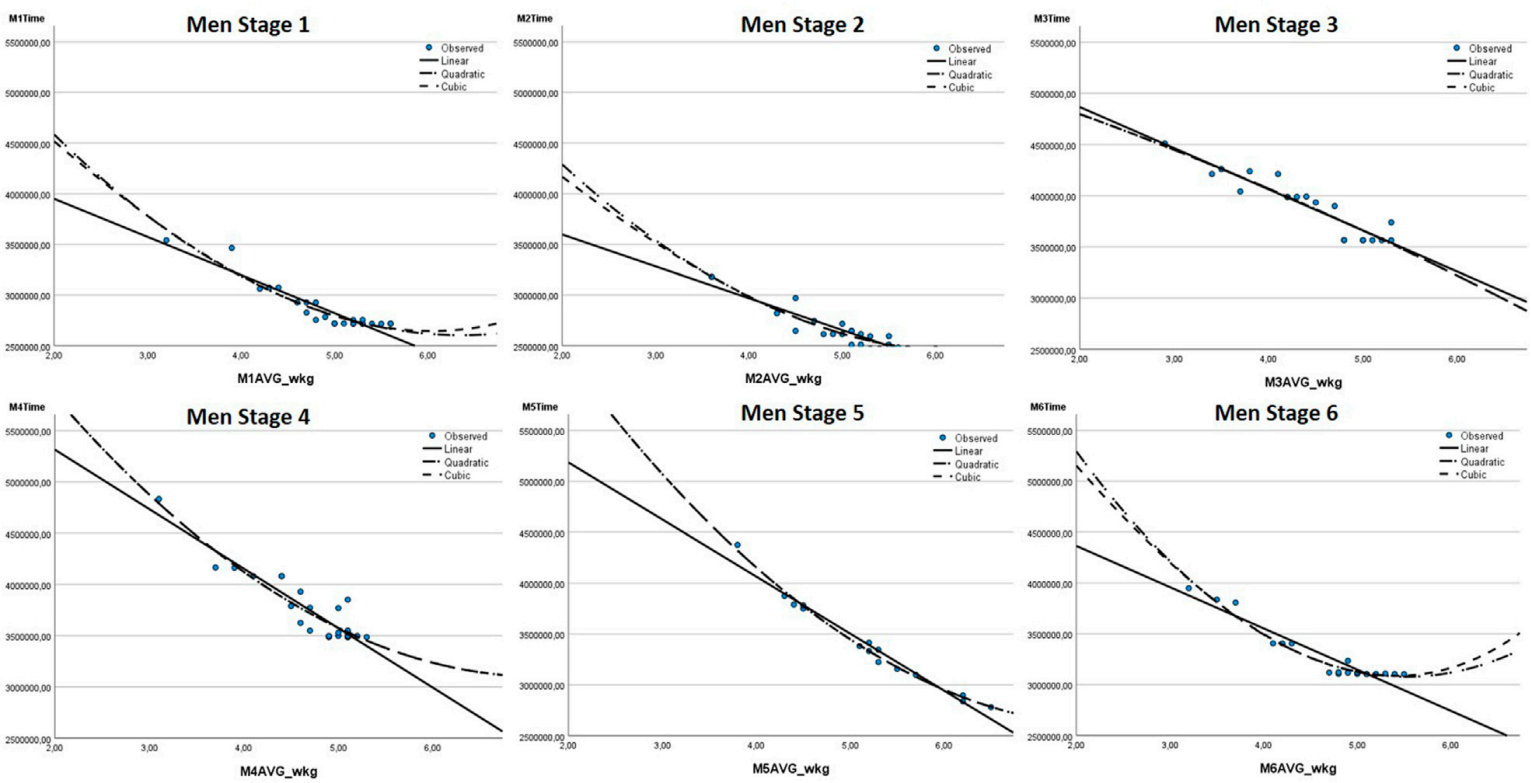
Scatterplot and fitted linear, quadratic and cubic relationship of relative power output (W/kg) and result (riding time in milliseconds) for men’s stages.

In road cycling, riders must overcome the forces of gravity, rolling resistance, and aerodynamic drag to move forward (Kyle, 2003). In particular, at a speed above 40 kph - which is exceeded in most professional races - the interaction of these three forces causes a cubic relationship between the power to be produced by the rider and the resulting speed, respectively riding time over a defined distance (Kyle, 2003). As mixed-reality platforms like Zwift seek to simulate speed as realistically as possible, the relationship between the performance parameters considered and the race outcome was visualized to gain initial insights on whether the cubic relationship also applies to mixed-reality competitions. In Figures 3 and 4, the relationship between the relative power output (in watts per kg body weight) and the competition result (riding time in milliseconds) is shown in scatterplots for the six stages of the women and men.

Using the example of the relationship between the relative power output and the competition result, the scatterplots of the respective stages show that the cubic graph fits better to the observed data points than the linear or the quadratic graph. Furthermore, comparable cubic patterns can be seen in particular for the other power output parameters (absolute and relative) and in an attenuated form for body mass and height.

### Data Analysis

In order to address the research question raised, the influence of the relative and absolute power over the complete duration, the relative power in different time intervals, and the rider’s body mass and height on the competition result were analyzed by performing regression analysis. Initially, we selected the appropriate type of regression analysis. Performing multiple regressions based on multiple parameters is not feasible because the variables depend on each other (absolute vs. relative power output) or represent overlapping time intervals. We tested considering multiple parameters. Thereby, the variance inflation factor was above 10 for almost all variables for all stages for both women and men, which is strong evidence for multicollinearity and should therefore be avoided (Hair et al., 2016). For this reason, the regression analyses are performed separately for all parameters that are specified in the research question.

Since the scatterplots in Figures 3 and 4 indicate a cubic relationship between the parameters and the competition results, third-order polynomial regressions are performed (see Eq. 1; Stimson et al., 1978). Polynomial regressions are a type of multiple regression, and therefore the assumptions of 1) no multicollinearity, 2) independence of residuals, 3) homoscedasticity, and 4) normal distribution of residuals must be considered. Multicollinearity often occurs when polynomial regressions are performed, because the first-, second-, and third-order polynomials of the independent variable are usually highly correlated. Therefore, it is not uncommon to have standardized regression coefficients of < −1 or > +1. To attenuate the effects of multicollinearity and improve interpretability of regression results, the included variables were mean-centered before running the polynomial regressions (Dalal and Zickar, 2012). Accordingly, all regressions are based on Eq. 1:

$$Y=b_{0}+b_{1}\times x+b_{2}\times x^{2}+b_{3}\times x^{3}+e$$ (1)

In Eq. 1, Y is the outcome variable (here: riding time in milliseconds), x is the mean-centered independent variable (predictor; 1) absolute power over the entire distance, 2) relative power over the entire distance, 3) maximum relative power over 20 min, 4) maximum relative power over 5 min, 5) maximum relative power over 30 s, 6) maximum relative power over 15 s, 7) body mass and 8) body height of a rider). b0 is the *Y*-intercept of the regression surface, while b1, b2, and b3 are the estimated coefficients of the predictor’s respective polynomial (first-, second-, third-order), and e is the random error component.

While multicollinearity is addressed via the mean centering function prior to running the regression analyses, the other assumptions are tested during the regression analysis to ensure the validity of the results. With regard to the second assumption (the independence of the residuals), the autocorrelation of the residuals is determined by performing the Durbin-Watson test (Allen, 1997). The test statistic values can thereby range from 0 to 4, with 0 indicating a perfectly positive autocorrelation, 4 indicating a perfectly negative autocorrelation, and 2 indicating no autocorrelation. Since the residuals should ideally be independent in polynomial regression, a value of 2 is desirable, while values between 1 and 3 are acceptable (Field, 2013). The third assumption, homoscedasticity of the residuals, is used to verify that the regression model makes good predictions across all values. Therefore, it is checked whether the dispersion of the residuals is constant. Finally, the assumption of normal distribution of the residuals is assessed by histograms. However, potential violations of this assumption have little to no impact on the execution and interpretation of the polynomial regressions (Stimson et al., 1978; Lumley et al., 2002).

All regression analyses were performed on the basis of the data sets described in Tables 1 and 2 with IBM SPSS Statistics software version 26. The data sets include the performance data of all participants of the respective stage, since the measurement and disclosure of the performance data is a prerequisite for the execution of mixed-reality competitions. To finally obtain the best model fit, the explanatory power of the cubic polynomial regression was compared with the explanatory power of the linear and quadratic functions (Stimson et al., 1978). Compared to the cubic function, neither the linear nor the quadratic regression function achieved a higher overall explanatory power for the relative and absolute performance parameters and body mass and height. Therefore, only the results of the third-order polynomial regression analyses are reported in the following results section.

## Results

The results of the third-order polynomial regression analyses for the women’s and men’s Virtual Tour de France stages are summarized in Table 3. Compared to (multiple) linear regressions, polynomial regression analyses do not allow meaningful interpretation of the strength of individual regression coefficients since the polynomials depend on each other and can only be varied simultaneously (Stimson et al., 1978). Accordingly, polynomial regressions are to be evaluated based on global fit indices. The *R* ^2^ indicates to what extent the variance of the dependent variable (competition result as riding time on the stage) can be explained by the independent variable (regressor). The *p*-value of the F-statistic indicates the extent to which the calculated *R* ^2^ differs significantly from 0. In Table 3, regression results are highlighted in italics if they do not meet one or more of the validity criteria. This can happen if 1) the 95% significance level (*p* > 0.05) is not reached, 2) the Durbin-Watson test shows values <1 or >3, which would indicate autocorrelation, or on the basis of the histograms respectively the scatterplots 3) heteroscedasticity or 4) no normal distribution of the residuals is observed. Furthermore, the strongest predictors (largest *R* ^2^) for each women’s and men’s stage are highlighted in bold. All other regression results (non-italics and non-bold) also meet the validity criteria. Due to limited interpretability, standardized regression coefficients, associated *p*-values, regression functions, and confidence intervals are presented in Supplementary Material SA, SB. The effects of regressors 1) to 9) on the competition outcome are listed per stage in the same order in Table 3 as in the research question raised.

**TABLE 3:** Regression results of women’s and men’s stages.

Regressor		Women			Men		
		F Statistics	*R* ^2^	Durbin-Watson	F Statistics	*R* ^2^	Durbin-Watson
Stage 1	a) Watts	F(3,29) = 31.927, *p* < 0.001	0.744	1.543	F(3,20) = 67.618, *p* < 0.001	0.897	1.189
	b) W/kg	**F(3,29) = 35.774, *p* < 0.001**	**0.765**	**1.447**	**F(3,20) = 133.695, *p* < 0.001**	**0.945**	**1.895**
	c) W/kg 20m	F(3,26) = 25.351, *p* < 0.001	0.716	1.475	F(3,20) = 23.423, *p* < 0.001	0.745	1.241
	d) W/kg 5m	F(3,29) = 11.666, *p* < 0.001	0.500	1.078	F(3,20) = 6.050, *p* = 0.004	0.397	1.035
	e) W/kg 1m	*F(3,29) = 6.703, p = 0.001*	*0.348*	*0.574*	F(3,20) = 8.562, *p* = 0.001	0.497	1.557
	f) W/kg 30s	*F(3,29) = 6.354, p = 0.002*	*0.334*	*0.630*	F(3,20) = 15.503, *p* < 0.001	0.654	1.518
	g) W/kg 15s	*F(3,29) = 2.229, p = 0.106*	*0.103*	*0.329*	F(3,20) = 18.867, *p* < 0.001	0.700	1.717
	h) Body mass	*F(3,25) = 0.505, p = 0.682*	*0*	*0.216*	*F(3,20) = 0.253, p = 0.858*	*0*	*0.210*
	i) Body height	*F(3,24) = 1.177, p = 0.339*	*0.019*	*0.223*	*F(3,16) = 0.479, p = 0.701*	*0*	*0.367*
Stage 2	a) Watts	F(3,37) = 17.584, *p* < 0.001	0.554	1.309	F(3,21) = 19.444, *p* < 0.001	0.697	1.170
	b) W/kg	**F(3,37) = 105.864, *p* < 0.001**	**0.887**	**1.455**	**F(3,21) = 55.731, *p* < 0.001**	**0.872**	**2.465**
	c) W/kg 20m	F(3,37) = 48.403, *p* < 0.001	0.780	2.084	F(3,21) = 42.114, *p* < 0.001	0.837	2.475
	d) W/kg 5m	F(3,37) = 30.094, *p* < 0.001	0.686	1.738	F(3,21) = 13.689, *p* < 0.001	0.613	1.726
	e) W/kg 1m	F(3,37) = 18.815, *p* < 0.001	0.572	1.539	*F(3,21) = 9.307, p <0.001*	*0.509*	*0.966*
	f) W/kg 30s	F(3,37) = 19.434, *p* < 0.001	0.580	1.503	*F(3,21) = 15.658, p <0.001*	*0.647*	*0.815*
	g) W/kg 15s	F(3,37) = 15.781, *p* < 0.001	0.526	1.362	F(3,21) = 11.395, *p* < 0.001	0.565	1.080
	h) Body mass	*F(3,33) = 1.080, p = 0.371*	*0.007*	*0.188*	*F(3,20) = 2.998, p = 0.055*	*0.207*	*0.625*
	i) Body height	*F(3,29) = 3.070, p = 0.043*	*0.241*	*0.441*	*F(3,14) = 0.254, p = 0.857*	*0*	*0.134*
Stage 3	a) Watts	F(3,29) = 50.558, *p* < 0.001	0.823	1.974	F(3,16) = 16.618, *p* < 0.001	0.711	1.771
	b) W/kg	**F(3,29) = 67.180, *p* < 0.001**	**0.861**	**1.727**	**F(3,16) = 35.694, *p* < 0.001**	**0.846**	**1.612**
	c) W/kg 20m	F(3,25) = 33.765, *p* < 0.001	0.778	1.819	F(3,14) = 11.213, *p* = 0.001	0.643	1.283
	d) W/kg 5m	F(3,25) = 8.538, *p* < 0.001	0.447	1.126	F(3,14) = 9.017, *p* = 0.001	0.586	1.779
	e) W/kg 1m	F(3,25) = 21.279, *p* < 0.001	0.685	1.288	F(3,14) = 8.088, *p* = 0.002	0.556	1.198
	f) W/kg 30s	F(3,25) = 7.835, *p* = 0.001	0.423	1.171	F(3,14) = 6.773, *p* = 0.005	0.505	1.198
	g) W/kg 15s	F(3,25) = 5.097, *p* = 0.001	0.305	1.092	*F(3,14) = 2.550, p = 0.098*	*0.215*	*0.430*
	h) Body mass	*F(3,22) = 0.380, p = 0.768*	*0*	*0.608*	*F(3,15) = 0.746, p = 0.541*	*0*	*0.384*
	i) Body height	*F(3,20) = 1.226, p = 0.326*	*0.029*	*0.337*	*F(3,9) = 0.456, p = 0.720*	*0*	*0.388*
Stage 4	a) Watts	*F(3,33) = 25.102, p <0.001*	*0.668*	*0.995*	F(3,23) = 15.277, *p* < 0.001	0.622	1.408
	b) W/kg	**F(3,35) = 95.967, *p* < 0.001**	**0.882**	**1.633**	F(3,23) = 46.446, *p* < 0.001	0.840	1.533
	c) W/kg 20m	F(3,29) = 26.341, *p* < 0.001	0.704	1.935	**F(3,19) = 40.354, *p* < 0.001**	**0.843**	**1.249**
	d) W/kg 5m	F(3,29) = 14.539, *p* < 0.001	0.559	1.467	F(3,19) = 28.976, *p* < 0.001	0.792	1.311
	e) W/kg 1m	*F(3,29) = 1.981, p = 0.139*	*0.084*	*0.866*	*F(3,19) = 25.814, p <0.001*	*0.772*	*0.711*
	f) W/kg 30s	*F(3,29) = 2.295, p = 0.099*	*0.108*	*0.790*	F(3,19) = 31.525, *p* < 0.001	0.806	1.122
	g) W/kg 15s	*F(3,28) = 2.863, p = 0.055*	*0.153*	*0.798*	*F(3,19) = 17.853, p <0.001*	*0.697*	*0.837*
	h) Body mass	*F(3,32) = 0.370, p = 0.775*	*0*	*0.148*	F(3,21) = 5.121, *p* = 0.008	0.340	1.006
	i) Body height	*F(3,28) = 1.435, p = 0.254*	*0.040*	*0.389*	*F(3,17) = 0.279, p = 0.840*	*0*	*0.201*
Stage 5	a) Watts	F(3,29) = 27.596, *p* < 0.001	0.714	1.478	F(3,11) = 6.940, *p* = 0.007	0.560	1.245
	b) W/kg	**F(3,29) = 601.166, *p* < 0.001**	**0.983**	**1.507**	**F(3,11) = 469.376, *p* < 0.001**	**0.990**	2.070
	c) W/kg 20m	F(3,29) = 122.040, *p* < 0.001	0.919	1.792	F(3,11) = 16.755, *p* < 0.001	0.771	2.353
	d) W/kg 5m	F(3,29) = 31.751, *p* < 0.001	0.742	1.286	F(3,11) = 16.364, *p* < 0.001	0.767	2.021
	e) W/kg 1m	F(3,29) = 18.267, *p* < 0.001	0.618	1.088	F(3,11) = 11.989, *p* = 0.001	0.702	2.132
	f) W/kg 30s	F(3,29) = 16.346, *p* < 0.001	0.590	1.192	F(3,11) = 6.909, *p* = 0.007	0.559	2.115
	g) W/kg 15s	F(3,29) = 13.246, *p* < 0.001	0.534	1.062	*F(3,11) = 2.834, p = 0.087*	*0.282*	*1.423*
	h) Body mass	*F(3,26) = 0.786, p = 0.512*	*0*	*0.174*	*F(3,10) = 10.102, p = 0.002*	*0.677*	*0.882*
	i) Body height	*F(3,24) = 0.125, p = 0.944*	*0*	*0.070*	*F(3,7) = 2.212, p = 0.174*	*0.267*	*1.128*
Stage 6	a) Watts	F(3,25) = 21.232, *p* < 0.001	0.684	1.715	F(3,15) = 63.846, *p* < 0.001	0.913	2.339
	b) W/kg	**F(3,25) =59.397, *p* < 0.001**	**0.862**	**1.942**	**F(3,15) = 183.748, *p* < 0.001**	**0.968**	**2.873**
	c) W/kg 20m	*F(3,25) = 9.266, p <0.001*	*0.470*	*0.845*	F(3,15) = 41.259, *p* < 0.001	0.870	1.246
	d) W/kg 5m	*F(3,25) = 9.307, p <0.001*	*0.471*	*0.889*	*F(3,15) = 4.127, p = 0.026*	*0.343*	*0.925*
	e) W/kg 1m	F(3,25) = 7.308, *p* = 0.001	0.403	1.365	F(3,15) = 7.709, *p* = 0.022	0.528	1.164
	f) W/kg 30s	F(3,25) = 5.484, *p* = 0.005	0.325	1.042	F(3,15) = 4.755, *p* = 0.016	0.385	1.058
	g) W/kg 15s	*F(3,25) = 1.312, p = 0.293*	*0.032*	*0.308*	*F(3,15) = 4.734, p = 0.016*	*0.384*	*0.944*
	h) Body mass	*F(3,24) = 0.397, p = 0.756*	*0*	*0.208*	*F(3,14) = 0.832, p = 0.498*	*0*	*0.640*
	i) Body height	*F(3,22) = 1.099, p = 0.371*	*0.012*	*0.606*	*F(3,12) = 0.510, p = 0.683*	*0*	*0.526*

Note. W/kg, relative power in watt per kg body weight; Watts, absolute power in watts; W/kg 20m, relative peak power over 20 min; W/kg 5m, relative peak power over 5 min; W/kg 1m, relative peak power over 1 min; W/kg 30s, relative peak power over 30 s; W/kg 15s, relative peak power over 15 s; mass, rider’s body mass in kilogram; body height, rider’s body height in centimeter; *R* ^2^, coefficient of determination (variance explained); strongest predictor of stage result in bold; results that are not significant at the 5% level are marked in italics.

The stages of the Virtual Tour de France have varying characteristics. Stage one was held on a hilly course. 1.5 km before the finish, the last mountain classification had to be mastered, followed by a descent to the finish. In the women’s race, the results show that relative power output over the entire stage duration (W/kg) has the greatest influence on the race result (*R* ^2^ = 0.765; *p* < 0.001). Relative power is less important the shorter the time interval considered. In men’s race, relative power output over the entire stage (W/kg; *R* ^2^ = 0.945; *p* < 0.001) is also the strongest predictor of success. The relative performances over time intervals of 15 s (W/kg 15s; *R* ^2^ = 0.700; *p* < 0.001) and 30 s (W/kg 30s; *R* ^2^ = 0.654; *p* < 0.001) also still show a high explanatory power.

Stage two led over one high mountain (>500 m of elevation gain) in the middle of the stage and ended with a flat section. In the women’s race, the relative performances over the entire distance (W/kg; *R* ^2^ = 0.887; *p* < 0.001) and over 20 (W/kg 20m; *R* ^2^ = 0.780; *p* < 0.001) and 5 min (W/kg 5m; *R* ^2^ = 0.686; *p* < 0.001) are particularly decisive for the race outcome. This result indicates that it was important to get over the mountain in the first group, while in the final part of the race, sprinting skills were less critical as the peloton was split into small groups. The men’s race developed in a similar way. Here the relative performance over the entire distance (W/kg; *R* ^2^ = 0.872; *p* < 0.001) also explains most of the success.

Stage three was an undulating course with a slightly hilly finish. In the women’s race, the strongest predictor is relative power over the entire distance (W/kg; *R* ^2^ = 0.861; *p* < 0.001). In the men’s race, relative power over the entire duration (W/kg; *R* ^2^ = 0.846; *p* < 0.001) is also the strongest predictor.

Stage four led over two smaller and two medium-high mountains (>100 m of elevation gain), with the last medium-high mountain about 3 km from the finish. In the women’s race, relative performance over the entire distance (W/kg; *R* ^2^ = 0.882; *p* < 0.001) is the strongest predictor, while shorter time intervals of the relative peak power (<5 min) do not significantly affect the competition result. However, the progression in the men’s race was different. Here, a comparatively large group sprinted for the win, which is indicated by the fact that relative peak power over 30 s (W/kg 30s; *R* ^2^ = 0.806; *p* < 0.001) is almost as critical for success as the strongest predictor relative peak power over 20 min (W/kg 20m; *R* ^2^ = 0.843; *p* < 0.001). Notably, body mass (*R* ^2^ = 0.340; *p* = 0.008) significantly affects competition results.

The fifth stage, after a short flat section, led up to a mountain top finish, where a difference in altitude of over 1,000 m had to be overcome. For both women (*R* ^2^ = 0.983; *p* < 0.001) and men (*R* ^2^ = 0.990; *p* < 0.001), relative power output over the entire distance is the strongest predictor of success due to the height of the mountain. For both genders, relative peak power over 1 minute still has a relatively high explanatory power (women: *R* ^2^ = 0.618; *p* < 0.001; men: *R* ^2^ = 0.702; *p* = 0.001). Overall, this is also reflected in the race progressions, which included a few accelerations on the uphill, but it was particularly important to keep relative peak power high to defend the advantage gained all the way to the finish. Remarkably, despite the mountainous course profile, body mass alone has no effect on race performance, neither in women (*p* = 0.512), nor in men (presence of autocorrelation due to Durbin-Watson statistic <1).

The sixth and final stage followed a slightly hilly route, with the last kilometers being flat. In the women’s race, relative peak power over the entire stage (W/kg; *R* ^2^ = 0.862; *p* < 0.001) is the strongest predictor of success and the explanatory power decrease constantly for shorter peak power intervals. In the men’s race, a bunch sprint occurred for the win. Here, relative power over the entire stage (W/kg; *R* ^2^ = 0.968; *p* < 0.001) is the strongest predictor for the competition results, while relative peak power interval of 1 minute (W/kg 1m; *R* ^2^ = 0.528; *p* = 0.022) also has a notable impact.

Overall, it appears that relative power output over the entire stage can explain the highest proportion of the variance in the riding time, and thus success in the race, for both women and men. However, the explanatory power (*R* ^2^) ranges between 77% and 98% for women and between 84% and 99% for men depending on the stage. Accordingly, for some stages, other performance indicators (regressors) explain success almost as well as - or in individual cases even better than (see men’s stage 4) - relative power output over the entire stage, which will be addressed in the following discussion.

## Discussion

Previous studies have investigated the relevance of absolute and relative power output (Gallo et al., 2021; Lee et al., 2002) and peak power (Faria et al., 2005) for success by relating performance data measured in a laboratory setting to success in outdoor competitions that are not directly related to that data. This study advances such work by focusing on mixed-reality sports platforms to analyze performance data from competition in combination with the directly corresponding competition outcome. Following the relationship between the power output generated by a rider and the resulting speed in the real world, a cubic relationship was assumed for the regression analyses performed here to model the relationship between the performance data and the riding time of the avatar over a defined distance in the virtual world. The third-order polynomial regression analyses reveal that the assumed cubic relationship also applies to mixed-reality sports.

We contribute to sports physiology science by indicating that the (relative) in-competition power output is the strongest predictor of success in mixed-reality competitions. The explanatory power of the examined power output, body mass, and body height depends on the course characteristics and the progression of the race. The role of course characteristics is indicated by the fact that for flat stages, the explanatory power of absolute and relative power output over the entire duration are comparable (e.g., stage 1 women: R^2^watts = 0.744 vs. R^2^w/kg = 0.765; stage 1 men: R^2^watts = 0.897 vs. R^2^w/kg = 0.945; similar effects are observed for stages 3 and 6). For mountain stages, the explanatory power of relative power output over the entire distance is considerably higher than for absolute power output (e.g., stage 5 women: R^2^watts = 0.714 vs. R^2^w/kg = 0.983; stage 5 men: R^2^watts = 0.560 vs. R^2^w/kg = 0.990; similarly, for stage 4). Body mass alone is a significant predictor of athletic success only on the mountain stages (e.g., stage 4 men: R^2^body mass = 0.340). The effect of the progression of the race on the relevance of different performance parameters for the competition’s result can be seen in the fact that on identical courses in the women’s and men’s races, different performance parameters explained success substantially differently. For example, stage 1 of the women’s race was ridden offensively, and the winner was decided in the sprint of a leading group. This shows that the relative performance over the entire distance has the strongest explanatory power (R^2^w/kg = 0.765) and decreases with the observation of shorter time intervals (e.g., R^2^w/kg 5m = 0.500), since it is initially critical for success to be in the leading group. Stage 1 of the men was decided in a mass sprint. Here, the relative power over the entire distance (R^2^w/kg = 0.945) also has the highest explanatory power, which initially also decreases with shorter time intervals of the relative peak power (e.g., R^2^w/kg 5m = 0.397), but then increases again substantially for the shortest time intervals considered (R^2^w/kg 30s = 0.654; R^2^w/kg 15s = 0.700), which might represent the critical performance in the mass sprint. Body height cannot explain the success in any stage. This result is notable because participants are required to report their height and current body mass prior to the competition. Furthermore, the scatterplots and the polynomial regressions showed that in mixed-reality competitions, there is a cubic relationship between power output and speed (riding time over a defined distance). The cubic relationship is also present in the real world, where aerodynamic drag is especially important at high speeds, in addition to gravity and rolling resistance (Kyle, 2003). Although the body height of the athletes is considered in mixed-reality competitions, we could not observe a significant influence on the competition result. The frontal area of the bike on which the power output is performed on the smart trainer in the real world has no effect on the competition result. The reason for this is that the athletes cannot provide any information on this before the competition, and therefore it cannot be taken into account by the algorithms of the mixed-reality sports platform. In summary, on the one hand, relative power over a long period of time is especially critical for success when long mountains have to be overcome on the course or when the race is ridden very offensively, and the peloton is divided into many small groups of riders. On the other hand, relative peak power over a shorter period of time is increasingly relevant with shorter climbs, especially towards the end of the race or when the race is decided in a bunch sprint.

We can conclude that the power profile of a mixed-reality cycling race is more complex compared to the rather constant power profiles that are delivered during ergometer tests in a laboratory setting over a specific period of time (e.g., 30 min; Lee et al., 2002) or in a time trial (e.g., Padilla et al., 2000). Understanding the power profile and its effect on competition outcome allows the transfer of insights from studies. Internal factors such as specific training programs or altitude training can be used to improve athletes’ critical performance parameters (e.g., Jeukendrup and Martin, 2001). In addition, factors such as seating position (Hansen and Waldeland, 2008), gear ratio and pedaling cadence that affect the cycling economy (Faria et al., 2005), or nutritional strategy (Atkinson et al., 2003) may increase relevant performance in mixed-reality competition. The findings from the area of indoor cycling are also relevant, where in particular an effect of starting strategy (Mattern et al., 2001), time of day (Atkinson et al., 2005), warm-up (Munro et al., 2017), recovery duration (Glaister et al., 2005), and precooling strategy (Quod et al., 2008) on athletic performance has been proven. It has already been demonstrated that the starting strategy in mixed-reality races is considerably different from that in road races, as the effort at the beginning of the race is significantly higher in mixed-reality races (Westmattelmann et al., 2021a).

**FIGURE 5:**
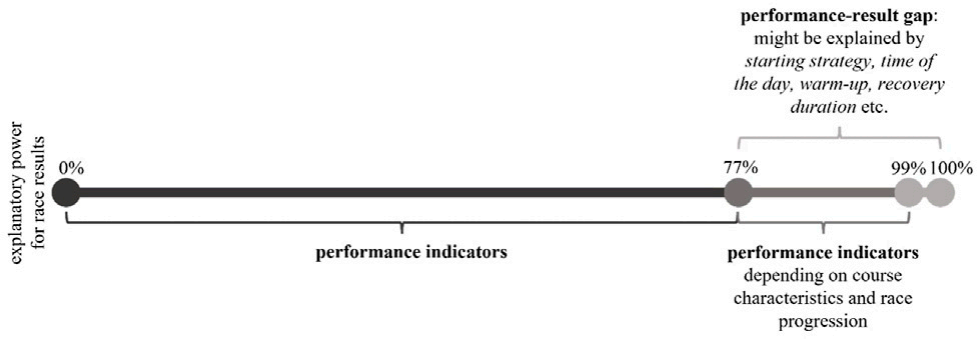
Performance-result gap exemplified by the Virtual Tour de France 2020.

This study revealed that specific performance parameters (power output) are the main predictors of success in mixed-reality competitions. Nevertheless, success in any stage of the Virtual Tour de France could not be explained entirely by only one of the performance parameters, resulting in a performance-result gap, which varies in size between 1% and 23% (Figure 5), depending on the characteristics of the course and the progression of the race. At first, it might be relevant at which point in time during the race the athletes produce certain power outputs. For example, a higher power output towards the end of the race or on a mountain could be more critical for success than if the power is produced at the beginning of the race or on a downhill segment. Furthermore, different external parameters could also explain the performance-result gap. Weight and aerodynamics of the real-world bike and the rider’s position are not directly relevant because the rider produces the performance while stationary on a smart trainer. In contrast, as aerodynamics, including slipstream effects, are simulated, and different virtual bikes and corresponding equipment have different weight and aerodynamic characteristics that resemble the real-world equipment, these can affect the competition result. Simulated slipstream effects, team strategy, or positioning in the peloton might also explain the performance-result gap (Van Erp et al., 2021b). It has to be mentioned that the rider’s positioning in the virtual race depends mostly on the rider’s power output because steering skills are not considered. Still, due to the absence of consideration of steering skills in mixed-reality competitions, such skills are likely to be less relevant for the competition result than in road races. While mechanical failures can be critical in road races, the risk of mechanical failures is much lower in mixed-reality competitions. For example, while the chain can still break, a punctured tire has no impact on the race. However, technical failures in the form of an unstable internet connection or crashed software are potential race-deciding aspects in mixed-reality competitions (Lazzari et al., 2020). Finally, the performance-result gap can also be explained by specific knowledge about the mixed-reality sports platform. A corresponding example from the Zwift platform represents the effective use of power-ups, which make the athlete lighter, more aerodynamic, or invisible to competitors for a certain period of time and can thus be crucial for the race result (Westmattelmann et al., 2021a).

### Practical Implications

Based on our findings, recommendations for action can be offered to different stakeholder groups in sports. First, our results provide athletes and coaches insights into which performance parameters are critical for success in mixed-reality sports. Accordingly, training programs can be tailored to prepare athletes to deliver the relevant critical performances. Since we provide extensive knowledge on the performance profile of mixed-reality competitions, they can also be systematically integrated into the preparation for real-world competitions to train in a competition-specific manner. This is especially beneficial when no competitions occur in the off-season, when factors such as unfavorable weather at outdoor competitions pose an increased risk of injury or infection.

Our analysis offers coaches, sports directors, or team managers a solid basis for decision-making to deploy athletes in mixed-reality competitions according to the respective course characteristics. For example, performance data from training or performance diagnostics can be compared with the required performance profiles of mixed-reality competitions. This allows the selection of athletes whose performance best matches the required performance profile. Furthermore, due to the wide availability of performance data, mixed-reality competitions offer scouts the opportunity to track and recruit athletes based on their performance and body composition.

For mixed-reality sports platform operators, it is valuable to show that physical performance is the most critical factor for success in the competitions on their platform. Accordingly, mixed-reality sports competitions host relevant and authentic athletic performances, which underlines the relevance of mixed-reality events. It is also notable that success is not solely determined by physical performance in mixed-reality competitions. This uncertainty about the outcome of the competition makes mixed-reality competitions interesting for spectators.

Finally, we provide key insights for sports federations on how to deal with mixed-reality sports platforms. Mixed-reality-specific factors might explain the performance-result gap, which raises the question for sports federations as to the extent to which they want to establish mixed-reality competitions as an independent discipline. In this regard, federations need to decide whether the mixed-reality events should be as realistic as possible or whether they deliberately enhance the presence of gamified elements and mechanisms. While discipline-specific skills and strategies are relevant in traditional cycling disciplines such as road cycling, mountain-biking, cyclo-cross, or track racing, the same is true for mixed-reality cycling. Mixed-reality cycling has the potential to introduce people with an affinity for esports to traditional sports and thus represents a promising extension to appeal to a younger target group. At the same time, it will be interesting to observe whether professionals from road cycling will also participate in mixed-reality cycling competitions in the future, as in the Virtual Tour de France, 2020b, or whether a separate professional scene will emerge.

### Limitations and Further Research

There are potential limitations to this study. First, the stages of the Virtual Tour de France were conducted in a decentralized manner due to the Covid-19 pandemic, so the organizers could not monitor the calibration of the smart trainer or the body mass and height of the riders to the same extent as that would have been the case if the event had been conducted in a centralized manner. In their analysis of the Virtual Tour de France, Westmattelmann et al. (2021b) showed that the performance data correspond to the performances of real-world professional races but also observes suspicious weight data. However, since minor differences in performance can be decisive for victory and defeat in competitive sports, the reliability of the measured data needs to be considered further so that, for example, incorrect calibrations of the smart trainer or power meter can be identified.

Second, the ability to apply the insights gained here from mixed-reality cycling races to road races might be limited. First, the competition mode differs, as the Virtual Tour de France, 2020b featured a team competition, which could affect the team strategy. In contrast, the Tour de France is decided based on the total riding time in an individual classification. In addition, in the Virtual Tour de France, 2020b, riders were swapped between stages, and the starting fields were also much smaller than in the real-world Tour de France. Nevertheless, the athlete population considered in this study is representative of the real-world Tour de France as the men’s Virtual Tour de France included almost exclusively professional teams and riders who also compete in the real Tour de France, while the women’s Virtual Tour de France included professionals.

Although essential aspects from the real world were simulated in the Virtual Tour de France such as elevation profiles and slipstream effect, there are still differences, such as power-ups or the non-inclusion of steering skills, as the avatar steers autonomously. Furthermore, some environmental factors, such as rain or crosswinds, which can affect the race result in road races, are not considered in mixed-reality races. Follow-up studies should aim to investigate the extent to which gamification elements such as power-ups are used strategically by riders and what effect the power-up use has on competition outcomes and thus explain the performance-result gap. In view of the increasing importance of mixed-reality sports, the results presented here nevertheless show a high relevance for sports physiology science (see Crivoi do Carmo et al., 2022). The wide-ranging data transparency required to conduct mixed-reality sport goes far beyond the data available from traditional sport and thus offers plenty of new research opportunities. Since the power output data of all participants of the Virtual Tour de France are available and were considered in this study, the effect of race performance data on the competition result can be quantified for the first time.

## Conclusion

This study shows that success in mixed-reality sports particularly depends on athletes’ physical performance. Exemplified by the performance data, rider’s body mass and height as well as results of the Virtual Tour de France, we demonstrate that the relative power output over the entire stage distance explains most of the variance in the riding time. However, depending on the course characteristics and the race progression, the explanatory power of the considered performance indicators differs in some cases considerably. While relative peak performances over a long period are most critical for success in races with longer climbs, relative peak performances of around 1 minute are critical for success in flatter races ending in a bunch sprint. The relevance of the relative performance indicates that the body mass of the riders has some importance. However, body mass alone can predict race success only occasionally when a mountain lies just before the finish. There was no significant effect for body height on the race result observed.

Overall, insights from sports science can be applied to train specific critical performances (e.g., Jeukendrup and Martin, 2001). Although physical performance is the main predictor of success in mixed-reality sport, a performance-result gap is identified. Depending on the stage, a maximum of 77%–98% of success in women and 84%–99% in men could be explained by performance indicators. Future studies will have to quantify which additional factors can explain this gap. Due to the complete availability of in-competition performance data, mixed-reality offers new research opportunities for sports science. Furthermore, mixed-reality sport has the potential to attract new audiences as an individual discipline and to complement traditional sport.

## Data Availability

The datasets analyzed for this study will be made available by the authors upon request.
