# Supplementary material for: The Performance-Result Gap in Mixed-Reality Cycling – Evidence From the Virtual Tour de France 2020 on Zwift
Source: Front Physiol. 2022 May 13;13:868902. doi: 10.3389/fphys.2022.868902 (PMC9136089; doi:10.3389/fphys.2022.868902)
Supplement: Supplementary file 1 [file DataSheet1.pdf]

Supplementary Material

Supplementary Material A. Regression coefficients women's stages.

	Regressor	ß$_i$	p$_i$	Regression function<br>$Y=b_0+b_1*x+b_2*x^2+b_3*x^3+e$	95% CI [LL, UL]
**Stage 1**	Watts	ß$_1$: -1.240<br>ß$_2$: 0.201<br>ß$_3$: 0.382	p$_1$: <0.001<br>p$_2$: 0.094<br>p$_3$: 0.099	Y=3198025.107-8448.632*watts+53.238*watts^2+1.511*watts^3	[-11212.661, -5684.604]<br>[-9.630, 116.106]<br>[-0.302, 3.323]
	W/kg	ß$_1$: -0.833<br>ß$_2$: 0.205<br>ß$_3$: 0.050	p$_1$: <0.001<br>p$_2$: 0.040<br>p$_3$: 0.790	Y=3214566.553-357537.266*w/kg+200146.597*w/kg^2+46191.472*w/kg^3	[-517179.518, -197895.013]<br>[9858.221, 390434.973]<br>[-305905.074, 398288.018]
	W/kg 20m	ß$_1$: -1.312<br>ß$_2$: 0.336<br>ß$_3$: 0.738	p$_1$: <0.001<br>p$_2$: 0.011<br>p$_3$: 0.007	Y=3227694.808-600229.600*w/kg+352906.764*w/kg^2+787201.913*w/kg^3	[-817305.073, -383154.127]<br>[86291.104, 619522.423]<br>[233220.914, 1341182.912]
	W/kg 5m	ß$_1$: -1.205<br>ß$_2$: 0.534<br>ß$_3$: 0.968	p$_1$: <0.001<br>p$_2$: 0.044<br>p$_3$: 0.019	Y=3217081.757-690794.040*w/kg+618635.523*w/kg^2+1246706.649*w/kg^3	[-997833.342, -383754.738]<br>[18821.707, 1218449.339]<br>[219801.240, 2273612.057]
	W/kg 1m	ß$_1$: -0.478<br>ß$_2$: 0.421<br>ß$_3$: -0.102	p$_1$: 0.160<br>p$_2$: 0.008<br>p$_3$: 0.763	Y=3187042.298-158377.464*w/kg+198099.033*w/kg^2-35533.326*w/kg^3	[-382974.464, 66219.537]<br>[55881.925, 340316.140]<br>[-274244.318, 203177.665]
	W/kg 30s	ß$_1$: 0.262<br>ß$_2$: 0.712<br>ß$_3$: -1.108	p$_1$: 0.507<br>p$_2$: 0.001<br>p$_3$: 0.017	Y=3173355.874+48985.890*w/kg+127166.216*w/kg^2-85740.630*w/kg^3	[-100157.616, 198129.397]<br>[53173.143, 201159.289]<br>[-154664.175, -16817.084]
	W/kg 15s	ß$_1$: -0.261<br>ß$_2$: 0.551<br>ß$_3$: -0.488	p$_1$: 0.406<br>p$_2$: 0.093<br>p$_3$: 0.276	Y=3173932.849-27264.684*w/kg+30137.600*w/kg^2-5823.462*w/kg^3	[-93427.865, 38898.497]<br>[-5380.868, 65656.067]<br>[-16619.528, 4972.604]
	Weight	ß$_1$: 0.261<br>ß$_2$: 0.081<br>ß$_3$: -0.271	p$_1$: 0.447<br>p$_2$: 0.833<br>p$_3$: 0.611	Y=3224510.703+9950.107*kg+368.634*kg^2-80.537*kg^3	[-16577.850, 36478.064]<br>[-3198.595, 3935.863]<br>[-402.401, 241.327]
	Height	ß$_1$: -0.352<br>ß$_2$: -0.025<br>ß$_3$: -0.008	p$_1$: 0.365<br>p$_2$: 0.898<br>p$_3$: 0.983	Y=3225534.985-10763.996*cm-112.295*cm^2-2.881*cm^3	[-34808.850, 13280.857]<br>[-1909.435, 1684.845]<br>[-276.546, 270.783]
**Stage 2**	Watts	ß$_1$: -0.905<br>ß$_2$: 0.064<br>ß$_3$: 0.184	p$_1$: <0.001<br>p$_2$: 0.554<br>p$_3$: 0.351	Y=3156514.188-8304.604*watts+15.262*watts^2+0.674*watts^3	[-11905.680, 4703.529]<br>[-36.504, 67.028]<br>[-0.772, 2.122]
	W/kg	ß$_1$: -0.847<br>ß$_2$: 0.264<br>ß$_3$: -0.103	p$_1$: <0.001<br>p$_2$: <0.001<br>p$_3$: 0.343	Y=3110550.046-404651.695*w/kg+242916.567*w/kg^2-84360.630*w/kg^3	[-505439.264, -303864.126]<br>[135903.193, 349929.941]<br>[-262332.613, 93611.353]
	W/kg 20m	ß$_1$: -0.956<br>ß$_2$: 0.161<br>ß$_3$: 0.106	p$_1$: <0.001<br>p$_2$: 0.055<br>p$_3$: 0.465	Y=3140762.179-532721.336w/kg+176589.814*w/kg^2+114213.865*w/kg^3	[-688797.683, -376644.989]<br>[-4087.948, 357267.575]<br>[-199253.556, 427681.285]
	W/kg 5m	ß$_1$: -0.940<br>ß$_2$: 0.188<br>ß$_3$: 0.057	p$_1$: <0.001<br>p$_2$: 0.107<br>p$_3$: 0.754	Y=3140427.475-383582.968*w/kg+91317.734*w/kg^2+18795.448*w/kg^3	[-512371.817, -254794.120]<br>[-20720.835, 203356.304]<br>[-102011.115, 139602.010]
	W/kg 1m	ß$_1$: -0.890<br>ß$_2$: 0.292<br>ß$_3$: 0.008	p$_1$: <0.001<br>p$_2$: 0.176<br>p$_3$: 0.975	Y=3128638.685-251571.598*w/kg+51206.984*w/kg^2+524.508*w/kg^3	[-341819.001, -161324.196]<br>[-34046.970, 126460.937]<br>[-33420.793, 34469.808]
	W/kg 30s	ß$_1$: -0.957<br>ß$_2$: 0.982<br>ß$_3$: -0.590	p$_1$: <0.001<br>p$_2$: 0.007<br>p$_3$: 0.106	Y=3077446.735-167276.694*w/kg+67907.666*w/kg^2-9517.775*w/kg^3	[-221112.680, -113440.709]<br>[19863.737, 115951.595]<br>[-21142.451, 2106.902]
	W/kg 15s	ß$_1$: -0.739<br>ß$_2$: 0.566<br>ß$_3$: -0.395	p$_1$: 0.001<br>p$_2$: 0.024<br>p$_3$: 0.225	Y=3084797.244-93148.782*w/kg+30488.318*w/kg^2-4724.687*w/kg^3	[-143605.483, -42692.081]<br>[4169.356, 56807.280]<br>[-12476.144, 3026.771]
	Weight	ß$_1$: -0.255<br>ß$_2$: 0.197<br>ß$_3$: 0.562	p$_1$: 0.532<br>p$_2$: 0.326<br>p$_3$: 0.205	Y=3126581.271-10828.243*kg+1288.408*kg^2-306.478*kg^3	[-45716.088, 24059.603]<br>[-1338.836, 3915.653]<br>[-176.218, 789.174]

	Height	β_1: 0.773<br>β_2: -0.046<br>β_3: 0.943	p_1: 0.019<br>p_2: 0.797<br>p_3: 0.007	Y=3102977.398+22046.955*cm-170.329cm^2-236.519*cm^3	[3917.140, 40176.771]<br>[-1511.829, 1171.171]<br>[-403.259, -69.780]
**Stage 3**	Watts	β_1: -0.604<br>β_2: 0.307<br>β_3: -0.139	p_1: <0.001<br>p_2: 0.049<br>p_3: 0.479	Y=4149483.386-6626.968*watts+63.883*watts^2+0.294*watts^3	[-9294.155, 3959.782]<br>[0.274, 127.492]<br>[-1.132, 0.544]
	W/kg	β_1: -0.667<br>β_2: 0.284<br>β_3: -0.093	p_1:<0.001<br>p_2: 0.067<br>p_3: 0.614	Y=4160439.860-493231.022*w/kg+245028.812*w/kg^2-51936.633*w/kg^3	[-645177.807, -341284.237]<br>[-18793.717, 508851.340]<br>[-260492.439, 156619.174]
	W/kg 20m	β_1: -0.379<br>β_2: 0.295<br>β_3: -0.337	p_1: 0.019<br>p_2: 0.046<br>p_3: 0.106	Y=4122734.790-389025.472*w/kg+545399.107*w/kg^2-634064.971*w/kg^3	[-709068.269, -68982.675]<br>[9955.437, 1080842.777]<br>[-1411896.646, 143766.703]
	W/kg 5m	β_1: -0.432<br>β_2: 0.371<br>β_3: -0.278	p_1: 0.233<br>p_2: 0.035<br>p_3: 0.463	Y=4093999.095-4511637.292*w/kg+1022502.396*w/kg^2-999728.430*w/kg^3	[-1212139.351, 308864.766]<br>[79583.090, 1965421.703]<br>[-3759772.644, 1760315.785]
	W/kg 1m	β_1: -0.323<br>β_2: 0.786<br>β_3: -0.762	p_1: 0.155<br>p_2: <0.001<br>p_3: 0.007	Y=4014097.134-157118.110*w/kg+450491.731*w/kg^2-251098.267*w/kg^3	[-377990.887, 63754.667]<br>[276758.324, 624225.139]<br>[-428061.626, -74134.909]
	W/kg 30s	β_1: -0.029<br>β_2: 0.478<br>β_3: -0.714	p_1: 0.939<br>p_2: 0.009<br>p_3: 0.085	Y=4095853.470-8107.875*w/kg+87379.516*w/kg^2-45500.935*w/kg^3	[-225358.469, 209142.726]<br>[23406.376, 151352.655]<br>[-97788.277, 6786.407]
	W/kg 15s	β_1: -0.212<br>β_2: 0.469<br>β_3: -0.510	p_1: 0.543<br>p_2: 0.033<br>p_3: 0.207	Y=4107372.333-40129.820*w/kg+35180.029*w/kg^2-8333.177*w/kg^3	[-174215.783, 93956.143]<br>[3123.082, 67236.977]<br>[-21591.087, 4924.734]
	Weight	β_1: -0.290<br>β_2: -0.220<br>β_3: 0.249	p_1: 0.464<br>p_2: 0.419<br>p_3: 0.582	Y=4256498.058-27032.452*kg-4148.604*kg^2+481.511*kg^3	[-102305.987, 48241.083]<br>[-14588.007, 6290.798]<br>[-1307.305, 2270.328]
	Height	β_1: -0.506<br>β_2: 0.024<br>β_3: 0.136	p_1: 0.121<br>p_2: 0.932<br>p_3: 0.771	Y=4141882.332-19366.071*cm+138.329*cm^2+60.209*cm^3	[-50687.301, 11955.160]<br>[-3184.386, 3461.045]<br>[-366.404, 486.822]
**Stage 4**	Watts	β_1: -0.614<br>β_2: 0.325<br>β_3: -0.093	p_1: 0.002<br>p_2: 0.043<br>p_3: 0.697	Y=4116297.887-6750.897*watts+66.843*watts^2-0.207*watts^3	[-10895.757, -2606.037]<br>[2.113, 131.573]<br>[-1.281, 0.867]
	W/kg	β_1: -0.816<br>β_2: 0.400<br>β_3: 0.162	p_1: <0.001<br>p_2: 0.001<br>p_3: 0.301	Y=4127024.869-611122.046*w/kg+337005.857*w/kg^2+89213.212*w/kg^3	[-756961.005, -465283.086]<br>[139488.265, 534523.450]<br>[-83216.936, 261643.361]
	W/kg 20m	β_1: -1.084<br>β_2: 0.497<br>β_3: 0.636	p_1: <0.001<br>p_2: 0.003<br>p_3: 0.011	Y=4071370.267-959660.357*w/kg+622927.102*w/kg^2+720848.650*w/kg^3	[-1280623.488, -638697.225]<br>[232831.210, 1013022.994]<br>[181170.331, 1260526.968]
	W/kg 5m	β_1: -0.946<br>β_2: 0.253<br>β_3: 0.269	p_1: <0.001<br>p_2: 0.042<br>p_3:0.201	Y=4062513.575-738072.998*w/kg+380199.777*w/kg+380048.145*w/kg^3	[-1065730.300, -410415.695]<br>[15627.048, 744772.505]<br>[-214219.989, 974316.280]
	W/kg 1m	β_1: -0.053<br>β_2: 0.113<br>β_3: -0.308	p_1: 0.864<br>p_2: 0.564<br>p_3: 0.359	Y=4096512.641-31061.497*w/kg+77325.083*w/kg^2-140129.492*w/kg^3	[-398574.812, 336451.819]<br>[-193421.755, 348071.920]<br>[-447502.316, 167243.332]
	W/kg 30s	β_1: -0.271<br>β_2: 0.120<br>β_3: -0.164	p_1: 0.439<br>p_2: 0.500<br>p_3: 0.639	Y=4092442.152-73441.507*w/kg+25914.764*w/kg^2-12457.084*w/kg^3	[-264655.182, 117772.168]<br>[-51766.301, 103595.829]<br>[-66198.262, 41284.093]
	W/kg 15s	β_1: -0.455<br>β_2: 0.165<br>β_3: -0.009	p_1: 0.246<br>p_2: 0.329<br>p_3: 0.981	Y=4070251.736-74566.907*w/kg+15752.568*w/kg^2-188.866*w/kg^3	[-203364.643, 54230.830]<br>[-16726.378, 48231.515]<br>[-16528.403, 16150.672]
	Weight	β_1: 0.304<br>β_2: 0.097<br>β_3: -0.197	p_1: 0.411<br>p_2: 0.618<br>p_3: 0.610	Y=4155834.636+22787.698*kg+1035.235*kg^2-163.786*kg^3	[-32907.646, 78483.043]<br>[-3149.973, 5220.443]<br>[-812.568, 484.696]
	Height	β_1: 0.113<br>β_2: 0.343<br>β_3: 0.068	p_1: 0.717<br>p_2: 0.069<br>p_3: 0.829	Y=4033743.047+3770.455*cm+1181.547*cm^2+13.969*cm^3	[-17288.193, 24829.083]<br>[-100.270, 2463.364]<br>[-117.336, 145.274]
**Stage 5**	Watts	β_1: -0.868<br>β_2: 0.275<br>β_3: 0.070	p_1: <0.001<br>p_2: 0.007<br>p_3: 0.715	Y=3868765.876-13337.906*watts+107.988*watts^2+0.378*watts^3	[-19270.778, -7405.035]<br>[31.927, 184.050]<br>[-1.716, 2.472]

	W/kg	ß$_1$: -0.870<br>ß$_2$: 0.200<br>ß$_3$: -0.007	p_1: <0.001<br>p_2: <0.001<br>p_3: 0.919	$Y=3871737.780-699642.325*w/kg+278337.624*w/kg^2-69995.975*w/kg^3$	[-783804.178, -615480.472]<br>[171054.788, 385620.461]<br>[-147041.250, 133049.301]
	W/kg 20m	ß$_1$: -1.044<br>ß$_2$: 0.208<br>ß$_3$: 0.184	p_1: <0.001<br>p_2: <0.001<br>p_3: 0.128	$Y=3903673.002-994654.563*w/kg+319329.970*w/kg^2+255494.710*w/kg^3$	[-1226630.709, -762678.418]<br>[154110.026, 484549.914]<br>[-78423.865, 589413.285]
	W/kg 5m	ß$_1$: -1.141<br>ß$_2$: 0.132<br>ß$_3$: 0.362	p_1: <0.001<br>p_2: 0.164<br>p_3: 0.039	$Y=3926301.733-1040360.782*w/kg+158787.751*w/kg^2+330020.573*w/kg^3$	[-1349512.292, -731209.273]<br>[-68828.574, 386404.077]<br>[17019.581, 643021.566]
	W/kg 1m	ß$_1$: -1.088<br>ß$_2$: 0.208<br>ß$_3$: 0.305	p_1: <0.001<br>p_2: 0.105<br>p_3: 0.189	$Y=3878709.109-639264.220*w/kg+108976.273*w/kg^2+81725.991*w/kg^3$	[-896411.214, -382117.226]<br>[-24296.909, 242249.455]<br>[-42650.329, 206102.311]
	W/kg 30s	ß$_1$: -0.967<br>ß$_2$: 0.322<br>ß$_3$: 0.119	p_1: <0.001<br>p_2: 0.102<br>p_3: 0.665	$Y=3865634.615-433043.433*w/kg+86637.220*w/kg^2+10946.002*w/kg^3$	[-612391.290, -253695.576]<br>[-18171.072, 191445.511]<br>[-40213.689, 62105.693]
	W/kg 15s	ß$_1$: -0.869<br>ß$_2$: 0.513<br>ß$_3$: -0.129	p_1: <0.001<br>p_2: 0.051<br>p_3: 0.696	$Y=3826415.086-303885.024*w/kg+86556.615*w/kg^2-5784.725*w/kg^3$	[-447809.687, -159960.360]<br>[-294.554, 173407.784]<br>[-35808.307, 24238.857]
	Weight	ß$_1$: 0.205<br>ß$_2$: -0.028<br>ß$_3$: 0.083	p_1: 0.650<br>p_2: 0.896<br>p_3: 0.860	$Y=3978556.631+19429.847*kg-407.813*kg^2+104.961*kg^3$	[-67542.401, 106402.095]<br>[-6748.162, 5932.536]<br>[-1109.929, 1319.852]
	Height	ß$_1$: 0.070<br>ß$_2$: -0.113<br>ß$_3$: -0.026	p_1: 0.871<br>p_2: 0.591<br>p_3: 0.952	$Y=3922036.330+4998.935*cm-1146.932*cm^2-20.563cm^3$	[-57754.766, 67752.637]<br>[-5491.298, 3197.435]<br>[-724.073, 682.946]
**Stage 6**	Watts	ß$_1$: -0.741<br>ß$_2$: 0.544<br>ß$_3$: 0.326	p_1: 0.002<br>p_2: 0.004<br>p_3: 0.243	$Y=3519020.538-6238.278*watts+109.569*watts^2+0.816*watts^3$	[-9937.488, -2539.069]<br>[38.352, 180.786]<br>[-0.590, 2.221]
	W/kg	ß$_1$: -1.015<br>ß$_2$: 0.531<br>ß$_3$: 0.455	p_1: <0.001<br>p_2: <0.001<br>p_3: 0.003	$Y=3529744.185-517249.033*w/kg+383682.368*w/kg^2+245369.108*w/kg^3$	[-640766.259, -393731.808]<br>[256253.130, 511111.605]<br>[89262.886, 401475.330]
	W/kg 20m	ß$_1$: -0.676<br>ß$_2$: 0.431<br>ß$_3$: 0.674	p_1: 0.011<br>p_2: 0.016<br>p_3: 0.506	$Y=3547829.456-439439.504*w/kg+439185.901*w/kg^2+1733485.597*w/kg^3$	[-767481.720, -111397.288]<br>[90624.026, 787747.776]<br>[-356325.124, 703296.317]
	W/kg 5m	ß$_1$: -0.387<br>ß$_2$: 0.235<br>ß$_3$: -0.265	p_1: 0.180<br>p_2: 0.158<br>p_3: 0.397	$Y=3569741.557-329353.382*w/kg+409845.332*w/kg^2-580739*w/kg^3$	[-821678.348, 162971.585]<br>[-170625.770, 990316.435]<br>[-1969747.830, 808269.056]
	W/kg 1m	ß$_1$: -0.565<br>ß$_2$: 0.279<br>ß$_3$: -0.056	p_1: 0.047<br>p_2: 0.106<br>p_3: 0.853	$Y=3546931.938-224125.971*w/kg+142713.730*w/kg^2-16842.394*w/kg^3$	[-445464.262, -2787.679]<br>[-32813.789, 318241.249]<br>[-201618.317, 167933.529]
	W/kg 30s	ß$_1$: -0.215<br>ß$_2$: 0.441<br>ß$_3$: -0.496	p_1: 0.553<br>p_2: 0.013<br>p_3: 0.198	$Y=3511895.498-46149.307*w/kg+73524.497*w/kg^2-28907.141*w/kg^3$	[-204349.787, 112051.174]<br>[16827.217, 130221.777]<br>[-73911.283, 16097.001]
	W/kg 15s	ß$_1$: -0.647<br>ß$_2$: -0.044<br>ß$_3$: 0.359	p_1: 0.094<br>p_2: 0.868<br>p_3: 0.432	$Y=3610210.166-99477.782*w/kg-3424.019*w/kg^2+6500.880*w/kg^3$	[-217161.558, 18205.973]<br>[-45344.566, 38496.528]<br>[-10263.969, 23265.728]
	Weight	ß$_1$: 0.243<br>ß$_2$: -0.237<br>ß$_3$: -0.198	p_1: 0.495<br>p_2: 0.444<br>p_3: 0.668	$Y=3641668.629+13077.576*kg-1345.067*kg^2-72.908*kg^3$	[-25875.355, 52030.507]<br>[-4909.550, 2219.416]<br>[-419.238, 273.422]
	Height	ß$_1$: 0.081<br>ß$_2$: 0.037<br>ß$_3$: 0.252	p_1: 0.848<br>p_2: 0.860<br>p_3: 0.551	$Y=3551194.674+3052.189*cm+152.969*cm^2+72.781*cm^3$	[-29620.794, 35725.173]<br>[-1628.082, 1934.020]<br>[-176.548, 322.111]

Note. W/kg = relative power in watt per kg bodyweight; Watts = absolute power in watts; W/kg 20m = relative peak power over 20 minutes; W/kg 5m = relative peak power over 5 minutes; W/kg 1m = relative peak power over 1 minute; W/kg 30s = relative peak power over 30 seconds; W/kg 15s = relative peak power over 15 seconds; weight = rider's body weight in kilogram; height = rider's body height in centimeter; ß$_i$ = standardized regression coefficient; p_i = p-value; 95% CI = 95% confidence interval; LL and UL indicate the lower and upper limits of a confidence interval.

Supplementary Material B. Regression coefficients men's stages.

	Regressor	β_i	p_i	Regression function $Y=b_0+b_1*x+b_2*x^2+b_3*x^3+e$	95% CI [LL, UL]
Stage 1	Watts	β_1: -0.889<br>β_2: 0.531<br>β_3: 0.287	p_1: <0.001<br>p_2: <0.001<br>p_3: 0.166	$Y=2805038.679-4476.514*watts+35.487*watts^2+0.161*watts^3$	[-6094.397, -2858.630]<br>[19.750, 51.224]<br>[-0.073, 0.396]
	W/kg	β_1: -0.990<br>β_2: 0.837<br>β_3: 0.733	p_1: <0.001<br>p_2: <0.001<br>p_3: <0.001	$Y=2799643.284-399708.753*w/kg+332147.842* w/kg^2+178670.852*w/kg^3$	[-468423.601, -330993.905]<br>[213135.805, 451159.879]<br>[95154.632, 262187.073]
	W/kg 20m	β_1: -1.383<br>β_2: 1.819<br>β_3: 2.303	p_1: <0.001<br>p_2: 0.005<br>p_3: 0.004	$Y=2830561.367-830218.518*w/kg+939677.516*w/kg^2+789965.199*w/kg^3$	[-1336215.789, -524221.247]<br>[320957.947, 1558397.086]<br>[284258.001, 1295682.396]
	W/kg 5m	β_1: -0.377<br>β_2: 0.086<br>β_3: -0.299	p_1: 0.312<br>p_2: 0.823<br>p_3: 0.601	$Y=2848971.791-201712.883*w/kg+57194.845*w/kg^2-149903.550*w/kg^3$	[-607732.006, 204306.240]<br>[-468995.636, 583385.325]<br>[-738172.013, 438364.913]
	W/kg 1m	β_1: 0.074<br>β_2: 0.519<br>β_3: -0.834	p_1: 0.892<br>p_2: 0.004<br>p_3: 0.028	$Y=2772235.325+20008.088*w/kg+164201.546*w/kg^2-140580.008*w/kg^3$	[-171136.024, 211152.200]<br>[57990.204, 270412.888]<br>[-264569.791, -16590.225]
	W/kg 30s	β_1: 0.350<br>β_2: 0.695<br>β_3: -1.163	p_1: 0.263<br>p_2: <0.001<br>p_3: 0.002	$Y=2744595.524+46566.373*w/kg+47334.215*w/kg^2-20520.673*w/kg^3$	[-37775.958, 130908.703]<br>[26234.183, 68434.248]<br>[-32456.665, -8584.682]
	W/kg 15s	β_1: 0.670<br>β_2: 0.545<br>β_3: -1.289	p_1: 0.039<br>p_2: <0.001<br>p_3: <0.001	$Y=2727087.294+67011.261*w/kg+23050.116*w/kg^2-11006.484*w/kg^3$	[3801.589, 130220.934]<br>[12333.294, 33766.938]<br>[-16341.298, -5671.670]
	Weight	β_1: -0.102<br>β_2: -0.093<br>β_3: -0.125	p_1: 0.823<br>p_2: 0.759<br>p_3: 0.815	$Y=2878540.316-4645.259*kg-641.837*kg^2-70.857*kg^3$	[-47396.466, 38105.949]<br>[-4940.294, 3656.620]<br>[-694.998, 553.284]
	Height	β_1: -0.107<br>β_2: -0.493<br>β_3: 0.377	p_1: 0.834<br>p_2: 0.335<br>p_3: 0.621	$Y=2806533.150-2075.051*cm-1149.835*cm^2+60.707*cm^3$	[-22702.642, 18552.540]<br>[-3599.616, 1299.947]<br>[-194.392, 315.807]
Stage 2	Watts	β_1: -0.454<br>β_2: 0.304<br>β_3: -0.241	p_1: 0.079<br>p_2: 0.044<br>p_3: 0.391	$Y=2590013.004-2554.174*watts+35.773*watts^2-0.337*watts^3$	[-5429.603, 321.255]<br>[1.012, 70.534]<br>[-1.136, 0.462]
	W/kg	β_1: -0.806<br>β_2: 0.312<br>β_3: 0.086	p_1: <0.001<br>p_2: 0.047<br>p_3: 0.708	$Y=2608318.012-275845.880*w/kg+112229.804*w/kg^2+20218.255*w/kg^3$	[-385323.648, -166368.111]<br>[1453.969, 223005.639]<br>[-90518.964, 130955.473]
	W/kg 20m	β_1: -0.705<br>β_2: 0.273<br>β_3: -0.039	p_1: <0.001<br>p_2: 0.088<br>p_3: 0.861	$Y=2604154.418-336940.366*w/kg+218292.536*w/kg^2-29238.778*w/kg^3$	[-490647.986, -183232.746]<br>[-35571.211, 472156.282]<br>[-372396.118, 313918.562]
	W/kg 5m	β_1: -0.882<br>β_2: 0.483<br>β_3: -0.089	p_1: 0.004<br>p_2: 0.016<br>p_3: 0.787	$Y=2569050.769-376242.694*w/kg+346908.586*w/kg^2-58214.222*w/kg^3$	[-617324.008, -135161.379]<br>[70526.850, 623287.322]<br>[-501351.488, 384923.043]
	W/kg 1m	β_1: -0.870<br>β_2: 0.680<br>β_3: -0.269	p_1: 0.003<br>p_2: 0.088<br>p_3: 0.591	$Y=2568040.786-191180.602*w/kg+105017.052*w/kg^2-16836.439*w/kg^3$	[-311466.863, -70894.340]<br>[-17208.474, 227242.579]<br>[-80945.189, 47272.311]
	W/kg 30s	β_1: -0.631<br>β_2: 0.572<br>β_3: -0.404	p_1: 0.030<br>p_2: 0.016<br>p_3: 0.280	$Y=2553172.338-56603.995*w/kg+20779.914*w/kg^2-2755.619*w/kg^3$	[-107231.667, -5976.323]<br>[4354.342, 37205.487]<br>[-7924.308, 2413.069]
	W/kg 15s	β_1: -0.687<br>β_2: 0.296<br>β_3: -0.090	p_1: 0.041<br>p_2: 0.095<br>p_3: 0.792	$Y=2590314.402-49716.568*w/kg+6956.735*w/kg^2-332.965*w/kg^3$	[-97122.861, -2310.275]<br>[-1308.419, 15221.886]<br>[-2923.124, 2257.193]
	Weight	β_1: 1.092<br>β_2: -0.375<br>β_3: -0.972	p_1: 0.009<br>p_2: 0.106<br>p_3: 0.026	$Y=2691372.834+37560.801*kg-1766.605*kg^2-318.035*kg^3$	[10678.836, 64442.765]<br>[-3942.944, 409.735]<br>[-593.486, -42.584]
	Height	β_1: 0.408<br>β_2: 0.214<br>β_3: -0.381	p_1: 0.498<br>p_2: 0.597<br>p_3: 0.606	$Y=2537621.278+5348.884*cm+500.165*cm^2-71.050*cm^3$	[-11137.030, 21834.798]<br>[-1484.523, 2484.853]<br>[-359.459, 217.360]

Stage 3	Watts	ß1: -1.063<br>ß2: -0.151<br>ß3: 0.146	p1: 0.002<br>p2: 0.524<br>p3: 0.712	Y=3939967.762-5797.802*watts-11.468*watts²+0.087*watts³	[-9053.897, -2541.706]<br>[-48.757, 25.821]<br>[-0.404, 0.578]
	W/kg	ß1: -1.056<br>ß2: 0.059<br>ß3: 0.183	p1: <0.001<br>p2: 0.707<br>p3: 0.471	Y=3892604.501-456179.290*w/kg+32643.633*w/kg²-61909.356*w/kg³	[-624426.559, -287932.021]<br>[-148436.239, 213723.504]<br>[-115855.998, 239674.709]
	W/kg 20m	ß1: -1.003<br>ß2: 0.237<br>ß3: 0.303	p1: 0.009<br>p2: 0.228<br>p3: 0.431	Y=3830789.804-549018.239*w/kg+192331.236*w/kg²+197681.519*w/kg³	[-937775.908, -160260.570]<br>[-134633.179, 519295.651]<br>[-325384.568, 720747.605]
	W/kg 5m	ß1: -1.182<br>ß2: 0.123<br>ß3: 0.460	p1: 0.002<br>p2: 0.448<br>p3: 0.163	Y=3835648.390-772324.203*w/kg+147146.966*w/kg²+520872.096*w/kg³	[-1212201.446, -332446.961]<br>[-257148.320, 551442.253]<br>[-237734.392, 1279478.583]
	W/kg 1m	ß1: -1.077<br>ß2: 0.313<br>ß3: 0.182	p1: 0.003<br>p2: 0.521<br>p3: 0.304	Y=3805130.416-375316.682*w/kg+79517.774*w/kg²+19738.218*w/kg³	[-596018.345, -154615.019]<br>[-179272.307, 338307.854]<br>[-119338.712, 158815.148]
	W/kg 30s	ß1: -1.073<br>ß2: 0.759<br>ß3: -0.229	p1: 0.005<br>p2: 0.287<br>p3: 0.776	Y=3765334.840-226793.118*w/kg+66874.767*w/kg²-5267.681*w/kg³	[-371398.699, -82187.537]<br>[-62576.358, 196325.892]<br>[-44140.913, 33605.551]
	W/kg 15s	ß1: -0.906<br>ß2: -0.605<br>ß3: 0.992	p1: 0.044<br>p2: 0.495<br>p3: 0.338	Y=3890062.386-142057.174*w/kg-27669.451*w/kg²+8586.563*w/kg³	[-279814.314, -4300.033]<br>[-112424.245, 57085.342]<br>[-9988.778, 27161.905]
	Weight	ß1: 0.386<br>ß2: 0.170<br>ß3: -0.437	p1: 0.485<br>p2: 0.825<br>p3: 0.670	Y=3816394.859+14390.716*kg+518.258*kg²-65.799*kg³	[-25828.689, 54610.121]<br>[-4385.143, 5421.659]<br>[-388.348, 256.751]
	Height	ß1: 0.542<br>ß2: -0.293<br>ß3: -0.246	p1: 0.513<br>p2: 0.635<br>p3: 0.825	Y=3789120.869+19881.259*cm-1798.819*cm²-129.954*cm³	[-46093.806, 85856.325]<br>[-10079.382, 6481.744]<br>[-1419.500, 1159.592]
Stage 4	Watts	ß1: -1.021<br>ß2: 0.504<br>ß3: 0.676	p1: <0.001<br>p2: 0.130<br>p3: 0.135	Y=3674042.574-7959.950*watts+67.364*watts²+0.867*watts³	[-11881.047, -4038.853]<br>[-21.303, 156.032]<br>[-0.289, 2.023]
	W/kg	ß1: -0.791<br>ß2: 0.164<br>ß3: -0.003	p1: <0.001<br>p2: 0.641<br>p3: 0.994	Y=3703902.853-498738.394*w/kg+101600.809*w/kg²-995.187*w/kg³	[-664541.027, -332935.762]<br>[-343824.892, 547026.509]<br>[-282559.162, 280568.788]
	W/kg 20m	ß1: -1.491<br>ß2: 0.698<br>ß3: 0.756	p1: <0.001<br>p2: <0.001<br>p3: <0.001	Y=3596485.262-1057511.054*w/kg+3009740.662*w/kg²-1692342.182*w/kg³	[-1430328.760, -684693.348]<br>[1554201.215, 4465280.109]<br>[874125.370, 2510558.995]
	W/kg 5m	ß1: -0.877<br>ß2: 0.781<br>ß3: 0.655	p1: <0.001<br>p2: 0.084<br>p3: 0.236	Y=3632588.412-424263.327*w/kg+228688.288*w/kg²+81159.344*w/kg³	[-632651.280, -215875.373]<br>[-33472.290, 490848.866]<br>[-57668.576, 219987.264]
	W/kg 1m	ß1: -0.709<br>ß2: 0.459<br>ß3: 0.000	p1: 0.003<br>p2: 0.005<br>p3: 0.999	Y=3597917.986-222144.559*w/kg+91549.453*w/kg²-11.693*w/kg³	[-360408.613, -83880.505]<br>[30831.181, 152267.724]<br>[-37394.435, 37371.049]
	W/kg 30s	ß1: -0.575<br>ß2: 0.531<br>ß3: -0.294	p1: 0.003<br>p2: <0.001<br>p3: 0.100	Y=3569918.932-114974.557*w/kg+51293.145*w/kg²-7243.635*w/kg³	[-186884.331, -43064.784]<br>[31984.563, 70601.727]<br>[-16018.439, 1531.168]
	W/kg 15s	ß1: -0.099<br>ß2: 0.348<br>ß3: -0.648	p1: 0.678<br>p2: 0.010<br>p3: 0.013	Y=3592792.110-15196.514*w/kg+22483.634*w/kg²-8636.309*w/kg³	[-90544.458, 60151.430]<br>[5994.198, 38973.070]<br>[-15263.781, -2008.838]
	Weight	ß1: 0.367<br>ß2: 1.041<br>ß3: 0.268	p1: 0.302<br>p2: 0.016<br>p3: 0.635	Y=3548249.424+13207.911*kg+3603.357*kg²+53.932*kg³	[-12739.624, 39155.447]<br>[757.083, 6449.630]<br>[-179.035, 286.900]
	Height	ß1: -0.377<br>ß2: 0.113<br>ß3: 0.247	p1: 0.482<br>p2: 0.640<br>p3: 0.644	Y=3595350.654-11131.534*cm+569.005*cm²+94.757*cm³	[-43803.292, 21540.224]<br>[-1953.200, 3091.210]<br>[-329.913, 519.427]
Stage 5	Watts	ß1: -1,379<br>ß2: -0.258<br>ß3: 0.809	p1: 0.005<br>p2: 0.313<br>p3: 0.106	Y=3449517.092-17581.006*watts-69.524*watts²+2.629*watts³	[-28798.334, -6363.679]<br>[-214.155, 75.107]<br>[-0.655, 5.913]
	W/kg	ß1: -0.900<br>ß2: 0.148<br>ß3: -0.097	p1: <0.001<br>p2: <0.001<br>p3: 0.150	Y=3345322.026-512643.698*w/kg+103863.672*w/kg²-40855.099*w/kg³	[-5911104.042, -434183.354]<br>[62789.007, 1449938.337]<br>[-99018.018, 17307.819]

	W/kg 20m	ß$_1$: -1.142<br>ß$_2$: 0.078<br>ß$_3$: 0.252	p$_1$: 0.003<br>p$_2$: 0.630<br>p$_3$: 0.456	Y=3359976.418-862343.902*w/kg+84615.675*w/kg^2+211664.252*w/kg^3	[-1355714.573, -368973.231]<br>[-291203.863, 460435.204]<br>[-391728.797, 815057.301]
	W/kg 5m	ß$_1$: -1.222<br>ß$_2$: 0.062<br>ß$_3$: 0.350	p$_1$: 0.002<br>p$_2$: 0.714<br>p$_3$: 0.309	Y=3350583.080-964280.339*w/kg+73152.713*w/kg^2+335448.779*w/kg^3	[-1476868.706, -451691.972]<br>[-355666.167, 501971.594]<br>[-357029.201, 1027926.759]
	W/kg 1m	ß$_1$: -0.932<br>ß$_2$: 0.296<br>ß$_3$: -0.040	p$_1$: 0.036<br>p$_2$: 0.138<br>p$_3$: 0.927	Y=3274793.377-640406.865*w/kg+336018.399*w/kg^2-33548.237*w/kg^3	[-1228863.987, -51949.742]<br>[-126511.068, 798547.867]<br>[-821705.280, 754608.807]
	W/kg 30s	ß$_1$: -1.185<br>ß$_2$: 0.174<br>ß$_3$: 0.469	p$_1$: 0.020<br>p$_2$: 0.395<br>p$_3$: 0.321	Y=3318148.290-725462.567*w/kg+149254.246*w/kg^2+259666.491*w/kg^3	[-1312471.250, -138453.883]<br>[-222037.958, 520546.450]<br>[-290038.653, 809371.636]
	W/kg 15s	ß$_1$: -0.839<br>ß$_2$: 0.182<br>ß$_3$: 0.285	p$_1$: 0.216<br>p$_2$: 0.461<br>p$_3$: 0.450	Y=3339824.358-441637.761*w/kg+121154.793*w/kg^2+109161.221*w/kg^3	[-1181381.240, 298105.719]<br>[-227560.876, 469870.462]<br>[-424527.510, 642849.952]
	Weight	ß$_1$: 0.581<br>ß$_2$: 0.345<br>ß$_3$: 0.158	p$_1$: 0.219<br>p$_2$: 0.076<br>p$_3$: 0.738	Y=3224791.414+35511.156*kg+2957.750*kg^2+89.278*kg^3	[-24834.988, 95857.300]<br>[-376.530, 6292.030]<br>[-488.570, 667.125]
	Height	ß$_1$: 0.510<br>ß$_2$: 0.014<br>ß$_3$: 0.202	p$_1$: 0.442<br>p$_2$: 0.961<br>p$_3$: 0.756	Y=3257167.562+23997.561*cm+61.775* cm^2+55.464*cm^3	[-45684.870, 93679.992]<br>[-2839.675, 2963.224]<br>[-350.652, 461.579]
**Stage 6**	Watts	ß$_1$: -0.978<br>ß$_2$: 0.592<br>ß$_3$: 0.528	p$_1$: <0.001<br>p$_2$: 0.004<br>p$_3$: 0.055	Y=3207055.564-5144.631*watts+42.972*watts^2+0.303*watts^3	[-6883.581, -3405.681]<br>[16.269, 69.674]<br>[-0.007, 0.613]
	W/kg	ß$_1$: -0.894<br>ß$_2$: 0.542<br>ß$_3$: 0.363	p$_1$: <0.001<br>p$_2$: <0.001<br>p$_3$: 0.015	Y=3196205.687-383505.158*w/kg+277128.880* w/kg^2+120216.421*w/kg^3	[-463391.043, -303619.274]<br>[178332.952, 375924.808]<br>[26609.979, 213822.864]
	W/kg 20m	ß$_1$: -1.060<br>ß$_2$: 0.844<br>ß$_3$: 0.939	p$_1$: <0.001<br>p$_2$: 0.005<br>p$_3$:0.010	Y=3230695.145-599899.472*w/kg+521786.597*w/kg^2+418973.257*w/kg^3	[-797047.775, -402751.168]<br>[178753.091, 864820.103]<br>[115498.268, 722448.246]
	W/kg 5m	ß$_1$: -0.605<br>ß$_2$: 0.331<br>ß$_3$: -0.045	p$_1$: 0.117<br>p$_2$: 0.109<br>p$_3$: 0.902	Y=3229505.668-524800.428*w/kg+547054.205*w/kg^2-89248.906*w/kg^3	[-1197205.901, 147605.044]<br>[-136922.651, 1231031.061]<br>[-1608667.574, 1430169.762]
	W/kg 1m	ß$_1$: -0.603<br>ß$_2$: 0.865<br>ß$_3$: -0.620	p$_1$: 0.054<br>p$_2$: 0.024<br>p$_3$: 0.207	Y=3130311.163-162408.812*w/kg+180702.964*w/kg^2-48510.967*w/kg^3	[-328058.468, 3240.844]<br>[27277.866, 334128.062]<br>[-126898.500, 29876.566]
	W/kg 30s	ß$_1$: -0.576<br>ß$_2$: 1.354<br>ß$_3$: -1.100	p$_1$: 0.086<br>p$_2$: 0.066<br>p$_3$: 0.191	Y= 3129475.509-93267.749*w/kg+75933.893*w/kg^2-12113.065*w/kg^3	[-201615.907, 15080.409]<br>[-5549.329, 157417.115]<br>[-30955.637, 6729.507]
	W/kg 15s	ß$_1$: -0.577<br>ß$_2$: 1.101<br>ß$_3$: -0.842	p$_1$: 0.094<br>p$_2$: 0.067<br>p$_3$: 0.241	Y=3153976.183-71742.317*w/kg+36348.309*w/kg^2-4258.026*w/kg^3	[-157167.583, 13682.949]<br>[-2928.477, 75625.094]<br>[-11690.628, 3174.575]
	Weight	ß$_1$: -0.447<br>ß$_2$: -0.392<br>ß$_3$: 0.119	p$_1$: 0.359<br>p$_2$: 0.581<br>p$_3$: 0.899	Y=3311616.654-23425.424*kg-2401.026*kg^2+52.121*kg^3	[-76391.749, 29540.902]<br>[-11517.767, 6715.714]<br>[-810.465, 914.707]
	Height	ß$_1$: -0.086<br>ß$_2$: -0.347<br>ß$_3$: -0.023	p$_1$: 0.877<br>p$_2$: 0.286<br>p$_3$: 0.969	Y=3198044.473-1858.850*cm-862.988*cm^2-4.138*cm^3	[-27548.601, 23830.902]<br>[-2545.574, 819.598]<br>[-230.260, 221.984]

Note. W/kg = relative power in watt per kg bodyweight; Watts = absolute power in watts; W/kg 20m = relative peak power over 20 minutes; W/kg 5m = relative peak power over 5 minutes; W/kg 1m = relative peak power over 1 minute; W/kg 30s = relative peak power over 30 seconds; W/kg 15s = relative peak power over 15 seconds; weight = rider's body weight in kilogram; height = rider's body height in centimeter; ß$_i$ = standardized regression coefficient; p_i = p-value; 95% CI = 95% confidence interval; LL and UL indicate the lower and upper limits of a confidence interval.
